# Orchestration of signaling by structural disorder in class 1 cytokine receptors

**DOI:** 10.1186/s12964-020-00626-6

**Published:** 2020-08-24

**Authors:** Pernille Seiffert, Katrine Bugge, Mads Nygaard, Gitte W. Haxholm, Jacob H. Martinsen, Martin N. Pedersen, Lise Arleth, Wouter Boomsma, Birthe B. Kragelund

**Affiliations:** 1 REPIN, Department of Biology, University of Copenhagen, Ole Maaloes Vej 5, DK-2200 Copenhagen N, Denmark; 2 Structural Biology and NMR Laboratory, Department of Biology, University of Copenhagen, Ole Maaloes Vej 5, DK-2200 Copenhagen N, Denmark; 3 Niels Bohr Institute, University of Copenhagen, Blegdamsvej 17, 2100 Copenhagen Ø, Denmark; 4 Department of Computer Science, University of Copenhagen, Universitetsparken 1, 2100 Copenhagen Ø, Denmark

**Keywords:** IDRs, IDPs, Signaling, NMR, SAXS, SLiM, Disorder, Structural biology, CIDER, IDDomainSpotter, Cytokine receptors, Transmembrane receptors

## Abstract

**Background:**

Class 1 cytokine receptors (C1CRs) are single-pass transmembrane proteins responsible for transmitting signals between the outside and the inside of cells. Remarkably, they orchestrate key biological processes such as proliferation, differentiation, immunity and growth through long disordered intracellular domains (ICDs), but without having intrinsic kinase activity. Despite these key roles, their characteristics remain rudimentarily understood.

**Methods:**

The current paper asks the question of why disorder has evolved to govern signaling of C1CRs by reviewing the literature in combination with new sequence and biophysical analyses of chain properties across the family.

**Results:**

We uncover that the C1CR-ICDs are fully disordered and brimming with SLiMs. Many of these short linear motifs (SLiMs) are overlapping, jointly signifying a complex regulation of interactions, including network rewiring by isoforms. The C1CR-ICDs have unique properties that distinguish them from most IDPs and we forward the perception that the C1CR-ICDs are far from simple strings with constitutively bound kinases. Rather, they carry both organizational and operational features left uncovered within their disorder, including mechanisms and complexities of regulatory functions.

**Conclusions:**

Critically, the understanding of the fascinating ability of these long, completely disordered chains to orchestrate complex cellular signaling pathways is still in its infancy, and we urge a perceptional shift away from the current simplistic view towards uncovering their full functionalities and potential.

Video abstract

## Background

The sequencing of the human genome and key observations from earlier research [1, 2], spurred the recognition of proteins and protein regions functioning without having three-dimensional folds. These intrinsically disordered proteins (IDPs) and regions (IDRs, collectively here referred to as IDPs), constitute around 30–40% of the human proteome [3] and perform key cellular and highly regulated processes such as transcription, translation and signaling [4–7]. IDPs show distinct sequence characteristics with higher frequencies of Pro, Glu, Ser, Gln, Lys, Ala, and Gly, and lower frequencies of Val, Leu, Ile, Trp and Cys. Hence, their sequences have particular properties of low hydrophobicity and high charge [8], resulting in their intrinsic inability to fold into a single, well-defined structure. Instead, IDPs take on ensembles of almost isoenergetic states, although these are far from random. For example, they may harbor lowly populated secondary structures that are relevant to binding [9] and to tuning the disordered ensemble [10], and alteration in the populations of these elements by e.g. mutations may lead to promotion of pathological states [11]. Interactions by IDPs are often mediated by short linear motifs (SLiMs), which are 2–12 residue sequence stretches that are typically recognized by patterns of conserved residues within an otherwise sparsely conserved sequence stretch [12]. SLiMs may overlap, and due to the structural plasticity of disordered regions, transiently exposed motifs and binding interfaces thus provide a trait of multispecificity to an otherwise simple binding site. Furthermore, flanking residues outside a SLiM and post translational modifications (PTMs) can tune affinity and add regulatory properties assisting in the spatiotemporal orchestration of multiple binding events [13, 14]. Indeed, PTMs are frequent in IDPs, in particular phosphorylations [15] and ubiquitylation [16], impacting functionalities and regulatory potential in several different ways. Thus, PTMs allow IDPs to function in rheostatic regulation [17] which are graded quantitative responses, and may also drive the formation or disruption of membrane-less organelles through processes known as liquid-liquid phase separation (LLPS) [18, 19]. Finally, the disordered nature of IDPs allows them to exploit diverse binding mechanisms by which they can fold-upon-binding [20–22], but also form complexes in which structural disorder is maintained to different degrees [23]. Here, the most extreme case is the formation of a completely disordered complex of functional relevance and extreme affinity [24].

### The class 1 cytokine receptors

**Fig. 1:**
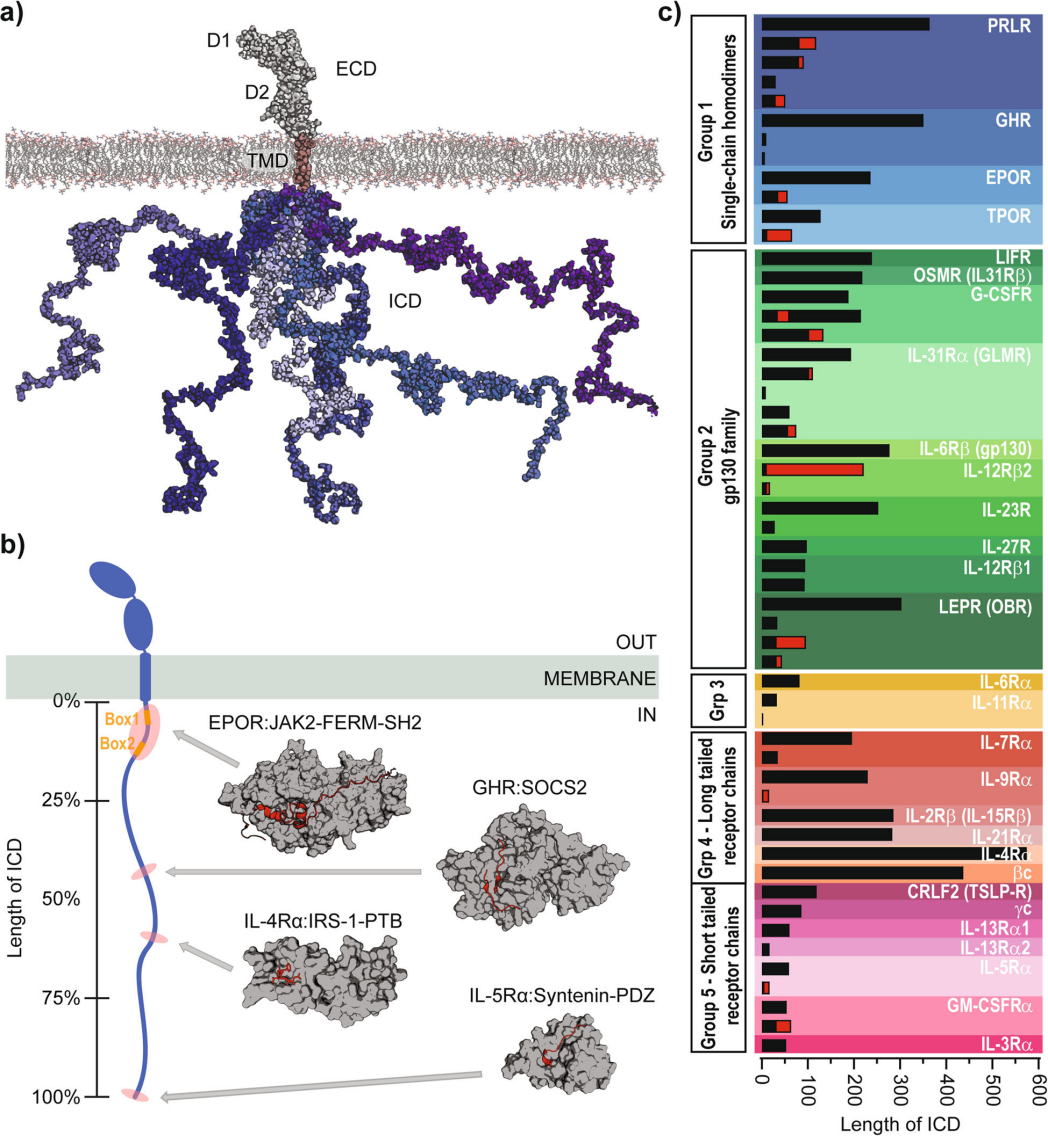
The C1CR family - structures and ICD isoforms. **a** Structural model of the full-length human PRLR [35] in the membrane, with the ECD in light grey, the TMD in pink and a total of six ICD chains shown in blue shades to represent its disordered conformational ensemble. **b** Left: A representative sketch of a prototypical C1CR (blue) in a membrane (green) having a long ICD with its length given in %. Box1 and Box2 are highlighted in orange. The red shades highlight the approximate extent and position of small ICD fragments of different C1CR-ICDs having their structure solved in complex with signaling proteins. Right: Three-dimensional structures of signaling proteins (grey surfaces) in complex with C1CR-ICD fragments (red cartoon and sticks), being the FERM-SH2 domain of JAK2 in complex with EPOR-ICD_279–334_ (pdb code 6E2Q) (top), SOCS2 in complex with GHR-ICD_591–603_ (pdb code 6I5J, 6I5N) (middle top), the PTB domain of IRS-1 in complex with IL-4Rα_490–500_ (pdb code 1IRS) (middle bottom) and the PDZ domain from syntenin in complex with IL-5Rα_417–420_ (pdb code 1OBZ) (bottom). The grey arrows point to the approximate positions of the ICD fragments (red shades) on the representative ICD sketch. **c** The 29 receptors of the C1CR family with an ICD and their isoforms. The length of the ICDs are to scale and red indicates the length of unique sequences differing from the long-form. The receptors are divided into 5 groups according to [33].

Structural disorder also exists in membrane proteins [25]. Besides being located in longer, disordered loops connecting transmembrane helices, such as in the sodium proton exchangers [26] and β*1*-adrenergic receptor [27], disorder prevails preferentially on the intracellular side [28], with membrane proteins having disordered N- and C-terminal tails of various length [29]. In fact, ~ 10% of the human membranome have disordered intracellular domains of > 100 residues classifying them as long disordered regions [29] and ~ 40% have disordered domains of > 30 residues [28]. For the subgroup of single-pass membrane proteins, an analysis of > 350 human sequences found disorder to be concentrated in the cytoplasmic domains [30], confirmed in very early work on gliotactin, a single-pass transmembrane receptor involved in cell adhesion [31]. An important family of single-pass transmembrane proteins with long disordered tails is the class 1 cytokine receptors (C1CRs) [32–34]. This family constitutes 40 members, which have been divided into five different groups based on their structural properties [33]. They all share having a tripartite structure characterized by a folded, extracellular domain (ECD) of various sizes and complexities, a single transmembrane helix (TMD), and an intracellular domain (ICD), also of varying length. Recently, the proportions of the different domains were exemplified by the a three-dimensional structural model of the prolactin receptor (PRLR); the first full-length structure of any cytokine receptor [35], Fig. 1a. C1CRs are characterized by the presence of two conserved cysteine bridges in the membrane distal fibronectin type III domain (D1) of the ECD, a WSxWS motif in the membrane proximal domain (D2) of the ECD, and two conserved sequence motifs in their ICD, Box1 and Box2 [32, 36]. The modular structures of the ECDs of the 40 receptors are well known and described broadly (see e.g. [32, 33]). Of the 40 receptors, 29 have an ICD (Fig. 1c), which all lack globular domains and intrinsic kinase activity. Signaling therefore critically depends on associated kinases such as Janus kinase 1–3 (JAK1, JAK2, JAK3) and tyrosine kinase 2 (TYK2) [37], Src kinases and mitogen activated protein kinases (MAPKs) [38]. Box1 is a proline-rich SLiM, onto which JAK are proposed to be constitutively bound [39–41]. Box2 is a less conserved region consisting of a sequence of hydrophobic residues, followed by negatively and then positively charged residues [42, 43]. The function of Box2 is unclear, but it is suggested to be important for efficient binding and activation of JAK1/2/3 and TYK2, possibly in cooperation with the region between Box1 and Box2 [39]. Recently, structures of complexes between JAK1/2 and TYK2 and a fragment of a cytokine receptor ICD were solved, namely of the erythropoietin receptor (EPOR) [44] (Fig. 1b), and of two class II cytokine receptors ICDs from the ﻿interferon-λ receptor 1 (﻿IFNLR1) [45] and interferon-α receptor chain 1 (IFNAR-1) [46], respectively. These complexes have revealed a common mode of interaction, where Box1 makes hydrophobic contacts to the FERM-domain of JAK1/2;TYK2 and a Glu of Box2 intercalates into the phospho-tyrosine binding pocket of the non-canonical SH2 domain of JAK1/2;TYK2, Fig. 1b. Src kinases are also suggested to bind to this region [47–50] most likely via a SH3-SH2 interaction.

C1CRs act in homo- and heteromeric dimers and oligomers, in some of which they share common receptor chains (IL-6Rβ (gp130), βc (IL-3Rβ) and γc (IL-2Rγ)). By binding the cytokine on the extracellular side, multiple common and receptor specific signaling events are initiated intracellularly with the main common pathways being activation of JAK/STAT, Src and MAPK signaling (for reviews on signaling, see e.g. [37, 38]). The receptors of the homodimeric group 1 (sometimes referred to as Type 1), constituting the EPOR, the growth hormone receptor (GHR), the thrombopoietin receptor (TPOR) and the PRLR, are considered to be structurally the simplest. This group has served as paradigmatic models of the family, and studies on the GHR have suggested that hormone binding leads to conformational changes in the receptor, including realignment and separation of the lower transmembrane domains, ultimately resulting in *trans*-phosphorylation of JAK2 and phosphorylation of the ICDs to initiate signaling [51, 52].

### Isoforms of C1CRs

Alternative splicing has been suggested to fine-tune the functions of IDPs [53, 54] and in accordance with this, functionally relevant alternative splicing has been shown to occur primarily in IDPs [53–56]. Furthermore, a large-scale computational analysis of alternative exons in human genes showed that alternatively spliced IDPs were highly enriched in interaction sites and regions modified by PTMs [53]. This has been suggested to provide a mechanism to rewire interaction networks by including or eliminating specific interaction motifs or binding sites [54]. Alternative splicing is further suggested to be important for maintaining tissue identity, as tissue-specific exons were shown to contain disordered regions enriched in PTMs and binding motifs that were central parts of tissue-specific interaction networks [53]. Many of the C1CRs also exist in different isoforms, but there are currently no reports available on their properties or numbers, or how their characteristics differ. For the PRLR, there are to date nine isoforms identified in humans, with four differing in their ECD [57–60], out of which one is an ECD soluble isoform [61]. The remaining five isoforms result from alternative mRNA splicing, and differ solely in length and amino acid composition of the ICD. These ICD isoforms constitute the predominant long form (LF, 622 residues), the intermediate form (IF, 349 residues), the short form 1a (SF1a, 376 residues), the short form 1b (SF1b, 288 residues) and the short form 1c (SF-1c, 309 residues), Fig. 1c. Compared to the LF, the IF, SF1a, SF1b and SF1c contain 13, 39, 3 and 24 unique C-terminal residues, respectively. The LF, IF, and SF1a all contain Box1 and Box2 motifs, while SF1b only contains Box1 [62–64]. Since activation of the JAK2/STAT5 pathway requires C-terminal tyrosine phosphorylation in the PRLR-ICD [65], this pathway is only activated by the LF due to lack of pYxxQ docking site motifs in the others [62, 64, 66, 67]. In contrast, the MAPK pathway is activated by the LF, SF1a, and SF1b [66, 68, 69], while the PI3K pathway is activated by both the LF [70, 71] as well as the short isoform in mice [66]. Heterodimerization of the LF with either of the shorter isoforms has been shown to inhibit activation of the JAK2/STAT5 pathway [62, 63]. The shortest isoform, PRLR-SF1b, has been shown to negatively regulate PRLR signaling [65]. Hence, isoform ratios need to be delicately balanced, and an increased ratio of short- to long forms has implications in breast- [72] and prostate [73] cancers. Lastly, since the different PRLR isoforms are expressed to different extents in different cell types and under different conditions [62–64], alternative splicing adds layers of regulation to PRLR signaling.

### Structural biology of C1CRs

Many C1CRs have been subjected to structural investigations, but remarkably, > 95% of the available structural information comes from the ECDs alone or in complexes with cytokine ligands. Furthermore, most ECD structures have been solved by X-ray crystallography, not providing information on dynamical parts. The understanding of the structure and function of single-pass TMDs is generally lacking behind [74]. For the C1CRs, structures of the TMDs have been solved almost exclusively on group 1 members, i.e. PRLR [35], EPOR [75, 76] and GHR [77] using detergent solubilized peptides and nuclear magnetic resonance (NMR) spectroscopy in both monomeric and dimeric states. However, monomeric structures have also been solved of the common receptor βc in bicelle membrane mimetics [78]. Still, no structural information is available for almost one fourth of the receptors, including the TPOR, interleukin (IL)-31Rβ, IL-31Rα, IL-12Rβ1, IL-12Rβ2, IL-27R, IL-9Rα, IL-11Rα, and the oncostatin-M-specific receptor (OSMR). Furthermore, only six structures are available of shorter fragments of ICDs in complex with signaling molecules, as exemplified by the juxtamembrane domain of the EPOR in complex with the FERM-SH2 domains of JAK2 [44], a 12-residue phospho-peptide from GHR-ICD in complex with SOCS2 [79], an 11-residue phospho-peptide of IL-4R-ICD with the phospho-tyrosine binding domain of the insulin receptor substrate (IRS)-1 [80], and an 8-residue peptide from IL-5Rα-ICD with the PDZ domain of syntenin [81], Fig. 1b. Drawn to scale on a representative ICD model (Fig. 1b), these binding sites take up only a minor part of the ICD, and leave a large area completely unexplored; not to mention the > 25 receptors for which no structures of any part of the ICD are available.

Using a combination of small-angle X-ray scattering (SAXS), NMR and circular dichroism (CD) spectroscopy we recently performed an extensive characterization of the ICD of GHR and PRLR, showing that the entire ICDs of the LFs of the human PRLR (PRLR-LF-ICD) and the human GHR (GHR-LF-ICD) are disordered, with only transiently populated helices [35, 82]. Moreover, the PRLR-LF-ICD interacts specifically with hallmark lipids of the inner membrane leaflet through three lipid interaction domains (LIDs), one overlapping with Box1 and Box2. The most membrane proximal of these was also identified in the GHR-LF-ICD suggesting similar roles in signaling. The only other reports on structural characterization of unbound ICDs of cytokine receptors available in the literature are on small synthetic peptides. One example is a 17-residue peptide from the IL-2Rβ-ICD covering a sorting signal (P^285^SKFFSQL^292^) and forming a type I β-turn [83]. Another example is a short peptide from PRLR-ICD containing the Box1 sequence (I^243^FPPVPGP^250^). The latter work suggested the presence of cis-trans isomerization of Pro248, the third proline of Box1 [84], supported by a suggested interaction with cyclophilin A (CypA), which co-immunoprecipitated with the first 76 intracellular residues of PRLR [85]. Besides for EPOR, PRLR and GHR, and the short 5–10 residue peptides from the IL-4R and IL-5Rα, the ICDs of the remaining 26 C1CRs of the family have not been studied at the atomic level, and only the PRLR-ICD and GHR-ICD have been studied in complete forms. This leaves a critical knowledge void inhibiting the understating of how C1CRs signal.

The present paper sets focus on the ICDs of the C1CRs and their structural disorder, and asks why disorder has evolved to manage versatility and fidelity in their signaling. We predict disorder in all human C1CRs and list all known isoforms that differ in the ICD. We analyze their primary structures, globally and locally, and, sequentially and experimentally analyze chain behavior identifying shared and unique characteristics across the family. We show that their sequences are brimming with SLiMs, conferring multispecificity to the chains. Instead of considering the ICDs as passive scaffolds for kinases, we put forward a more complex view of active orchestration via organizational and operational features left uncovered within their disorder.

## Materials and methods

### Proteins - expression and purification

Human PRLR-ICD^236–396^ was prepared as described in [82]. PRLR-SF1b-ICD was produced as a GST-tagged fusion protein containing a thrombin cleavage site. PRLR-SF1b-ICD was purified essentially following the procedure for PRLR-LF-ICD [82] with the following modifications: After inoculation of cells from overnight cultures in LB-medium, cells were left growing at 37 °C until OD_600_ = 0.8. The cells were subsequently centrifuged gently for 15 min at 2500 x g at 4 °C, the supernatant discarded, and the cells gently resuspended by swirling in 500 mL ^15^N- or ^13^C- and ^15^N-labeled M9-minimal media (1.5 g KH_2_PO4, 3.75 g Na_2_HPO_4_•2H_2_O, 0.5 g NaCl, 1 mM MgSO_4_, 0.5 ml M2 trace solution, 2 g ^13^C α-D-Glucose, 0.5 g ^15^NH_4_Cl) with 100 μg/ml Amp. The cell suspension was transferred back into the 5 L Erlenmeyer flask and left growing at 37 °C for 45 min after which protein expression was induced with 1 mM isopropyl β-D-1-thiogalactopyranoside (IPTG) for 3 h. The cells were harvested by centrifugation (20 min, 5000 x g, 4 °C) and stored at - 20 °C until thawed on ice and resuspended in 40 mL sonication buffer; 1x PBS (1.4 M NaCl, 27 mM KCl, 100 mM Na_2_HPO_4_, 18 mM KH_2_PO_4_, 10 x stock), 0.1% (v/v) Triton X-100 and one complete EDTA-free protease inhibitor cocktail tablet (Roche Diagnostics GmbH). The cells were sonicated on ice, using an UP400S Ultrasonic Processor, 4 × 30 s with 30 s rest between rounds at 90% amplitude. The cell extracts were centrifuged (25 min, 20,000 x g, 4 °C), the pellets discarded and the supernatants used for purification. The glutathione column (Glutathione Sepharose 4 Fast Flow, GE Healthcare) was prepared by washing with 20 column volumes (CVs) 1xPBS buffer pH 8 and the supernatant from sonication was incubated with the column material for 1 h at room temperature (RT) under gentle agitation. The column was washed with 20 CVs 1xPBS buffer pH 8 and GST-PRLR-SF1b-ICD was eluted using 10 CVs elution buffer (50 mM Tris-HCl pH 8, 10 mM reduced glutathione). The sample was dialyzed overnight at 4 °C under stirring against 1 L thrombin cleavage buffer (20 mM Tris-HCl, 150 mM NaCl pH 8.4) using a 6000–8000 MWCO dialysis membrane. One hundred units of thrombin (GE Healthcare) was added to the sample, which then incubated for 2 h at RT under gentle agitation. After cleavage, the sample was concentrated using centrifugal filters (3000 MWCO, Millipore) and applied to an analytical size exclusion column (Superdex 75 10/300 GL, GE Healthcare) equilibrated with 2 CVs of 50 mM Na_2_HPO_4_/NaH_2_PO_4_ pH 7.5, 150 mM NaCl and 0.1 mM DTT. The flowrate was 0.5 ml/min and PRLR-SF1b-ICD was eluted over 1.5 CV GHR-ICD-LF and PRLR-ICD-LF were preoduced as described in [35, 82].

### Lipids

Small unilamellar vesicles (SUVs) containing POPC/POPS and POPC were prepared as in [82].

### CD spectroscopy

Far-UV CD spectra were recorded on a Jasco-810 spectropolarimeter from 250 nm to 190 nm with a scan speed of 20 nm/min, bandwidth 1 nm, 2 s response time at 25 °C in a 1 mm quartz cuvette. Protein concentration was 19 μM in 10 mM NaH_2_PO_4_-NaOH, pH 7.4, 1 mM TCEP. The spectra were averaged over 10 scans with the corresponding spectrum of the buffer subtracted. The resulting spectra were smoothed using a fast Fourier transform, removing the highest frequencies in the spectrum.

### NMR spectroscopy

For backbone assignment, ^13^C-^15^N-labeled PRLR-SF1b-ICD was concentrated to 500 μM in 20 mM Na_2_HPO_4_/NaH_2_PO_4_ pH 7.3 or 7 M urea pH 7.3 (native and denatured conditions, respectively). The samples were added 10% (v/v) D_2_O, 5 mM tris (2-carboxyethyl) phosphine (TCEP), and 0.5 mM 2,2-dimethyl-2-silapentane-5-sulfonic acid (DSS) for referencing in a total volume of 350 μl. The pH was adjusted to 7.3 if needed and the samples were transferred to 5 mm Shigemi tubes. All backbone spectra were recorded at 5 °C on Varian INOVA 750- or 800-MHz (^1^H) spectrometers and backbone assignment accomplished from analyses of ^1^H-^15^N-HSQC [86], HNCACB [87], CBCA (CO) NH [88], and HNCO [89] spectra. Free induction decays were transformed and visualized using NMRPipe [90] and analyzed using CcpNmr Analysis [91]. Assignments were done manually. Transient secondary structure elements were identified by secondary chemical shifts (SCSs). These were calculated by subtracting the C^α^ and C′ chemical shifts for each residue in 7 M urea from those obtained in 20 mM Na_2_HPO_4_/NaH_2_PO_4_. The amount of transient α-helices was assessed as described [92]. Two series of ^1^H-^15^N-HSQC [86] spectra were recorded on ^15^N-PRLR-SF1b-ICD at 5 °C to analyze the relaxation times. The spectra for PRLR-SF1b-ICD were recorded with delay times of 10–1000 ms (T1) and 10–250 ms (T2) with two triplicates in each series. The relaxation decays were fitted to single exponentials and the relaxation times were determined using the CcpNmr Analysis software [91]. For CypA interaction studies, samples containing 100 μM ^15^N-labelled PRLR-SF1b-ICD or PRLR-LF-ICD with and without 85 μM human CypA (Sigma Aldrich) were prepared in 20 mM Na_2_HPO_4_/NaH_2_PO_4_ pH 7.3, 10% (v/v) D_2_O, 1 mM TCEP, 0.5 mM DSS. To accommodate slight differences in conditions of the different protein batches, all batches were thoroughly dialyzed against the same buffer before mixing the samples. ^1^H-^15^N-HSQC spectra were recorded on each sample at 5 °C and chemical shift changes were analyzed as combined amide chemical shift changes by δΔ_NH_ = ((δΔ_H_)^2^ + (0.154*δΔ_N_)^2^)^1/2^ [93].

### Small angle X-ray scattering

SAXS data were collected at the EMBL beamline P12 at Petra III in Hamburg, Germany [94]. SAXS data on PRLR-LF-ICD and GHR-LF-ICD (20 mM Na_2_HPO_4_/NaH_2_PO_4_ (pH 7.3), 10 times molar excess DTT) were collected at the PETRA III, P12 beamline (DESY synchrotron, Hamburg), following standard procedures. A series of different concentrations between 1 and 6 mg/mL were measured for each protein added 0, 75, 150 and 300 mM NaCl. The SAXS curves for PRLR-ICD-LF and GHR-ICD-LF were analyzed using the form-factor for a Gaussian random coil [95], together with a scaling factor for correcting the protein concentration and a constant to model the background. The fitting was done with the *minimize* method in optimization library in the *scipy* package for python 3 using the L-BFGS-B algorithm. Uncertainties in the fitting parameters were estimated from the diagonal of the inverse Hessian. Pair distance distribution functions were calculated using the BayesApp [96, 97] and using the GenApp server (https://genapp.rocks (visited on April 15, 2020)). In the case of PRLR-ICD-LF, a few data points were omitted at low-Q based on the Guinier analysis, but data from 0.0075 Å^-1^ and up was used in all cases. The Guinier analysis was also used to truncate the data for GHR-ICD so that only data above 0.0084 Å^-1^ was used. Due to the lower S/N, data above 0.3 Å^-1^ was also omitted from the analysis and the Lagrange multiplier was fixed to 10^14^, in order to ensure a stable solution with respect to data range and input parameters.

To calculate a reference *Rg* for PRLR-LF-ICD and GHR-LF-ICD, we used the power-law in Eq. 1, where *R*_*0*_ is a constant related to the persistence length of the polymer, N is the number of amino acids and *ν* is related to the nature of the polymer.

1 $$ Rg={R}_0{N}^{\nu } $$

For R_0_ and *ν*, we used the experimental parameters from Kohn and co-workers [98], determined based on chemically unfolded proteins, i.e. R_0_ = 1.927 Å and *ν* = 0.598, where the latter value is very close to the theoretical value of 0.588 of self-avoiding polymer chains.

### Disorder predictions, sequence alignments and bioinformatics

**Fig. 2:**
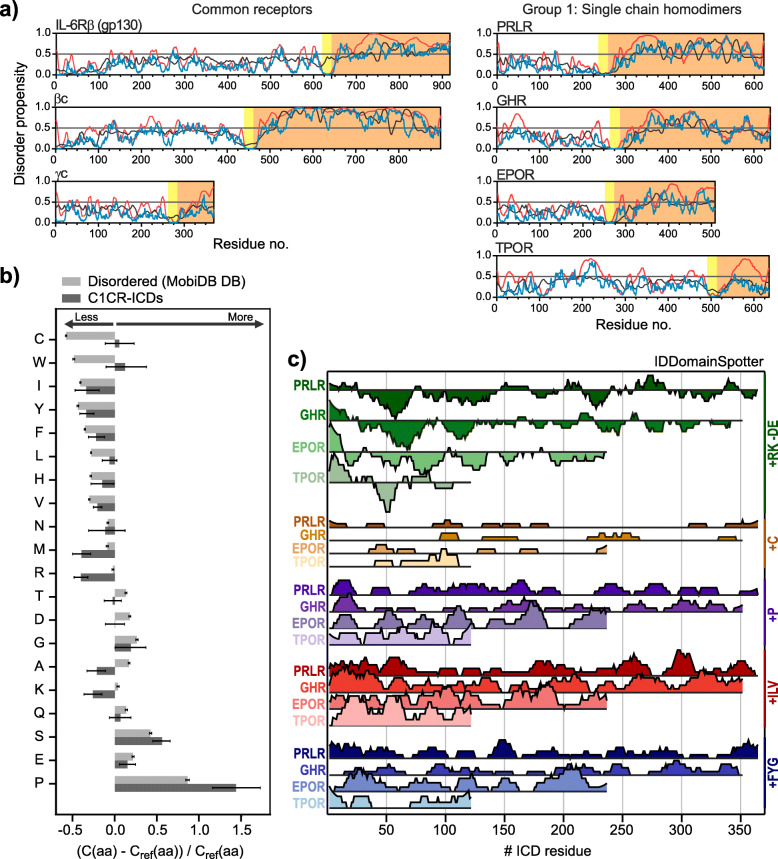
Intrinsic disorder and compositional bias of C1CR-ICDs. **a** Disorder prediction for C1CR common receptors and group 1. The disorder propensity, ranging from 0 to 1 was predicted using IUPred2A (blue), ANCHOR (black) and Pondr-fit VSL2 (red) and is plotted as a function of residue number. The boundaries between the ECD (white background), TMD (yellow background) and ICD (orange background) were predicted using TMHMM v. 2.0. The sequence numbering includes the signal peptide. **b** Fractional differences in composition between the C1CR-ICDs (dark grey) or a set of IDPs (light grey), and a set of folded proteins calculated for each amino acid type (see methods for details). Negative values denote that the amino acid is less frequent than in folded proteins, and positive values denote that the amino acid is more frequent than in folded proteins. The arrows indicate the directions of “more” abundant than in folded proteins, and “less” abundant than in folded proteins. The error bars indicate the 90% confidence interval of the estimated frequencies, calculated using a per-protein bootstrapping procedure with 1000 iterations [105]. **c** IDDomainSpotter profiles of group 1 C1CR-ICDs. Profiles display scores for +Arg,Lys-Asp,Glu (green), +Cys (brown), +Pro (purple), + Ile,Leu,Val (red) and + Phe,Tyr,Gly (blue) calculated over 15 residue windows for each of the ICDs

The original list of C1CRs was manually curated from the list of receptors in [33, 34], after which isoforms were extracted from Uniprot [99]. Disorder predictions for each of the sequences were conducted as described previously [13], and the sequences were analyzed by IDDomainSpotter [100], CIDER [101] and Weblogo3.0 [102] using standard setting and TMHMM was used to predict borders between domains [103]. The amino acid propensities for each of the sequences were calculated relative to an ordered reference statistic, as (C(aa) - C_ref(aa)) / C_ref(aa), where C(aa) is the frequency for the individual residue and C_ref(aa) is the frequency of the residue in the reference set. We calculated our ordered reference frequencies, C_ref(aa), from entries in the MobiDB database [104], using the curated *DB* set and selecting all protein regions labeled as ‘S’. The same set was used to calculate the frequencies for the Disorder category in Fig. 2b, this time selecting regions labeled as ‘D’. To validate the sensitivity to this choice, we also calculated the frequencies from the larger *Derived* MobiDB set, with qualitatively similar results. Finally, we verified the impact of homology reduction on these frequencies by reweighting each protein by the number of other entries in its UniRef50 class, but again observed similar results, and homology reduction was therefore omitted for the remaining calculations. The error bars in Fig. 2b indicate the 90% confidence interval of the estimated frequencies, calculated using a per-protein bootstrapping procedure with 1000 iterations [105].

SLiMs and phosphorylation sites were predicted in the longest isoforms of group 1 receptors (PRLR-FL, GHR-FL, EPOR-FL and TPOR1). SLiMs were predicted using the ELM resource [106] and filtered based on taxonomic context (*Homo sapiens*) and cellular compartment (cytosol) with a probability cut-off of 100. Modification sites were excluded from analysis. The iGPS 1.0 algorithm [107] was used for prediction of in vivo phosphorylation sites. Only instances above a medium threshold [107] was included. Confirmed SliMs and phosphorylation sites were manually curated.

## Results and discussion

### Disorder in C1CRs

The ICDs of the C1CRs have been very sparsely studied structurally, likely because of their high expected abundance of disorder. Moreover, many of the receptors exist in several different alternatively spliced versions, some of which differ in the ICDs. To provide an overview of the ICD isoforms, we conducted a survey of known isoforms of the entire family (Fig. 1c and Table 1). We followed the grouping of the receptors made based on structure and evolution [33, 34], but excluded receptors absent in humans and/or for which no ICD could be annotated. Furthermore, IL-2Rα and IL-15Rα were excluded as they lack the structural hallmarks of the family and likely belong to a separate family [108]. This left us with a total of 29 different receptors, distributed with four members in group I (single-chain homodimers), ten members in group II (the gp130 family, not counting receptors binding ancestral cytokines), two members in group III (soluble α-chains, leaving out four receptors without ICDs (IL-27Rβ, ciliary neurotrophic factor receptor subunit α (cntfRα), cytokine receptor-like factor 1 (CRLF1), and IL-12Rβ), six members in group IV (long-tailed receptor chains) and seven members in group V (short-tailed receptor chains), Table 1. The 29 receptors provided a total of 54 ICD isoforms distributed across all groups. Approximately 40% of the receptors (12 receptors; leukemia inhibitory factor receptor (LIFR), OSMR, IL-6Rβ (gp130), IL-27R, IL-6Rα, IL-2Rβ, IL21R, IL4Rα, βc (IL-3Rβ), CRLF2, γc (IL-2Rγ), IL-13Rα1, IL-13Rα2, and IL3Rα) only had one ICD isoform, and three of these were the common receptors IL-6Rβ (gp130), βc (IL-3Rβ) and γc (IL-2Rγ). For the remaining 16 receptors, up to five different isoforms could be identified and nine of the receptors with ICDs > 200 residues had a short isoform < 50 residues. A total of 16 isoforms had unique sequences, typically > 10 residues with an average length of 25 ± 16 residues, and the longest unique sequence of 67 residues belonged to the LEPR isoform C. The average length of the longest ICD isoforms was 188 residues (group I), 210 residues (group 2), 82 residues (group 3), 335 residues (group 4) and 65 residues (group 5) (Table 1). IL-4Rα had the longest ICD of 575 residues (Table 1). Thus, the C1CR-ICDs are generally long and some have isoforms with unique sequences of considerable length.

**Table 1:** Overview of isoforms of the cytokine class 1 receptor family differing in the ICD

Class 1 Cytokine Receptors with Intracellular Domains										
	Name^#^	Iso form	Alternative Names	Uniprot Identifier	ICD sequence (Bold: Identical in all ICD isoforms, Italics: Unique sequence)	Residues^a^	Unique start	Unique end	ICD length	ICD start
GROUP 1 - HOMODIMERIC RECEPTORS	**PRLR**	1,2^b^	PRLRfl^1^ ΔS1^2^	p16471-1	**KGYSMVTCIFPPVPGPKIKGFDAHLLE**KGKSEELLSALGCQDFPPTSDYEDLLVEYLEVDDSEDQHLMSVHSKEHPSQGMKPTYLDPDTDSGRGSCDSPSLLSEKCEEPQANPSTFYDPEVIEKPENPETTHTWDPQCISMEGKIPYFHAGGSKCSTWPLPQPSQHNPRSSYHNITDVCELAVGPAGAPATLLNEAGKDALKSSQTIKSREEGKATQQREVESFHSETDQDTPWLLPQEKTPFGSAKPLDYVEIHKVNKDGALSLLPKQRENSGKPKKPGTPENNKEYAKVSGVMDNNILVLVPDPHAKNVACFEESAKEAPPSLEQNQAEKALANFTATSSKCRLQLGGLDYLDPACFTHSFH	622^1^ 521^2^			364	259
		4	SF1a	p16471-4	**KGYSMVTCIFPPVPGPKIKGFDAHLLE**KGKSEELLSALGCQDFPPTSDYEDLLVEYLEVDDSEDQHLMSVHSKEHPSQG*DPLMLGASHYKNLKSYRPRKISSQGRLAVFTKATLTTVQ*	376	338	376	118	259
		5	I	p16471-5	**KGYSMVTCIFPPVPGPKIKGFDAHLLE**KGKSEELLSALGCQDFPPTSDYEDLLVEYLEVDDSEDQHLMSVHSKEHPSQ*EREQRQAQEARDS*	349	337	349	91	259
		6,8^c^	SF1b^6^ Δ4-SF1b^8^	p16471-6	**KGYSMVTCIFPPVPGPKIKGFDAHLLE***VTP*	288^6^ 217^8^	286^6^ 215^8^	288^6^ 217^8^	30	259^6^ 188^8^
		9	SF1c	p16471-9	**KGYSMVTCIFPPVPGPKIKGFDAHLLE***DRLCTPGRCCVSTGLTDLDYSCST*	309	286	309	51	259
	**GHR**	1,4^d^	GHRfl^1^ GHRd3^4^	p10912-1	**SKQQ**RIKMLILPPVPVPKIKGIDPDLLKEGKLEEVNTILAIHDSYKPEFHSDDSWVEFIELDIDEPDEKTEESDTDRLLSSDHEKSHSNLGVKDGDSGRTSCCEPDILETDFNANDIHEGTSEVAQPQRLKGEADLLCLDQKNQNNSPYHDACPATQQPSVIQAEKNKPQPLPTEGAESTHQAAHIQLSNPSSLSNIDFYAQVSDITPAGSVVLSPGQKNKAGMSQCDMHPEMVSLCQENFLMDNAYFCEADAKKCIPVAPHIKVESHIQPSLNQEDIYITTESLTTAAGRPGTGEHVPGSEMPVPDYTSIHIVQSPQGLILNATALPLPDKEFLSSCGYVSTDQLNKIMP	638^1^ 616^4^			351	288^1^ 266^4^
		2	GHRtr GHR1-279	p10912-2	**SKQQ***SSSSKD*	297	292	297	10	288
		3	GHR1-277	p10912-3	**SKQQ***KEN*	294	292	294	7	288
	**EPOR**	F	FL	p19235-1	**SHRRALKQKIWPGIPSPESEFEGLFTTHKGNFQ**LWLYQNDGCLWWSPCTPFTEDPPASLEVLSERCWGTMQAVEPGTDDEGPLLEPVGSEHAQDTYLVLDKWLLPRNPPSEDLPGPGGSVDIVAMDEGSEASSCSSALASKPSPEGASAASFEYTILDPSSQLLRPWTLCPELPPTPPHLKYLYLVVSDSGISTDYSSGDSQGAQGGLSDGPYSNPYENSLIPAAEPLPPSYVACS	508			236	273
		T	Truncated	p19235-3	**SHRRALKQKIWPGIPSPESEFEGLFTTHKGNFQ***VGGLVVPSVPGLPCFLQPNCRPL*	328	306	328	56	273
	**TPOR**	1	C-mpl-P	p40238-1	**RWQFPAHYR**RLRHALWPSLPDLHRVLGQYLRDTAALSPPKATVSDTCEEVEPSLLEILPKSSERTPLPLCSSQAQMDYRRLQPSCLGTMPLSVCPPMAESGSCCTTHIANHSYLPLSYWQQP	635			121	515
		2	C-mpl-K	p40238-2	**RWQFPAHYR**YRPR*QAGDWRWTRWSRTCKQAFLVRSVTPDLRPPPVRTYGFALPARHLWDSPRLLTL*	579	523	579	66	514
GROUP 2 - gp130 FAMILY	**LIFR**	1		P42702-1	YRKREWIKETFYPDIPNPENCKALQFQKSVCEGSSALKTLEMNPCTPNNVEVLETRSAFPKIEDTEIISPVAERPEDRSDAEPENHVVVSYCPPIIEEEIPNPAADEAGGTAQVIYIDVQSMYQPQAKPEEEQENDPVGGAGYKPQMHLPINSTVEDIAAEEDLDKTAGYRPQANVNTWNLVSPDSPRSIDSNSEIVSFGSPCSINSRQFLIPPKDEDSPKSNGGGWSFTNFFQNKPND	1097			239	859
	**OSMR** (IL-31Rβ)	1	OSMRfl IL-31Rβ	Q99650-1	KSQWIKETCYPDIPDPYKSSILSLIKFKENPHLIIMNVSDCIPDAIEVVSKPEGTKIQFLGTRKSLTETELTKPNYLYLLPTEKNHSGPGPCICFENLTYNQAASDSGSCGHVPVSPKAPSMLGLMTSPENVLKALEKNYMNSLGEIPAGETSLNYVSQLASPMFGDKDSLPTNPVEAPHCSEYKMQMAVSLRLALPPPTENSSLSSITLLDPGEHYC	979			218	762
	**G-CSFR**	1	G-CSF-R1	Q99062-1	**CCSPNRKNPLWPSVPDPAHSSLGSWVPTIMEE**DAFQLPGLGTPPITKLTVLEEDEKKPVPWESHNSSETCGLPTLVQTYVLQGDPRAVSTQPQSQSGTSDQVLYGQLLGSPTSPGPGHYLRCDSTQPLLAGLTPSPKSYENLWFQASPLGTLVTPAPSQEDDCVFGPLLNFPLLQGIRVHGMEALGSF	836			188	649
		3	G-CSF-R3	Q99062-3	**CCSPNRKNPLWPSVPDPAHSSLGSWVPTIMEE***LPGPRQGQWLGQTSEMSRALTPHPCVQ*DAFQLPGLGTPPITKLTVLEEDEKKPVPWESHNSSETCGLPTLVQTYVLQGDPRAVSTQPQSQSGTSDQVLYGQLLGSPTSPGPGHYLRCDSTQPLLAGLTPSPKSYENLWFQASPLGTLVTPAPSQEDDCVFGPLLNFPLLQGIRVHGMEALGSF	863	680	707	215	649
		4	GCSF-R4	Q99062-4	**CCSPNRKNPLWPSVPDPAHSSLGSWVPTIMEE**DAFQLPGLGTPPITKLTVLEEDEKKPVPWESHNSSETCGLPTLVQTYVLQGDPRAVSTQPQSQSGTSDQ*AGPPRRSAYFKDQIMLHPAPPNGLLCLFPITSVL*	783	750	783	134	650
	**IL-31Rα** (GLMR)	1, 2, 6,12	V3^2^ GPL745^12^	Q8NI17-1^1^ Q8NI17-2^2^ Q8NI17-6^6^ 8NI17-12^12^	**VAYGLKKPN**KLTHLCWPTVPNPAESSIATWHGDDFKDKLNLKESDDSVNTEDRILKPCSTPSDKLVIDKLVVNFGNVLQEIFTDEARTGQENNLGGEKNGYVTCPFRPDCPLGKSFEELPVSPEIPPRKSQYLRSRMPEGTRPEAKEQLLFSGQSLVPDHLCEEGAPNPYLKNSVTAREFLVSEKLPEHTKGEV	732^1^ 764^2^ 622^6^ 745^12^			194	539^1^ 571^2^ 429^6^ 552^12^
		3,5	v4 v1	Q8NI17-3^3^ Q8NI17-5^5^	**VAYGLKKPN**KLTHLCWPTVPNPAESSIATWHGDDFKDKLNLKESDDSVNTEDRILKPCSTPSDKLVIDKLVVNFGNVLQEIFTDEARTGQENNLGGEKNG*TRILSSCPTSI*	662^3^ 681^5^	639^3^ 639^5^	649^3^ 649^5^	111	552^3^ 571^5^
		9	GPL560, short	Q8NI17-9^9^	**VAYGLKKPN**	560			9	552
		10	GPL610	Q8NI17-10^10^	**VAYGLKKPN**KLTHLCWPTVPNPAESSIATWHGDDFKDKLNLKESDDSVNTEDRI*RARYQA*	611	593	598	60	552
		11	GLP626	Q8NI17-11^11^	**VAYGLKKPN**KLTHLCWPTVPNPAESSIATWHGDDFKDKLNLKESDDSVNTEDRI*KGSELGTKLKFKPLISLDCAF*	626	593	613	75	552
	**IL-6Rβ** (gp130)	1,3		P40189-1^1^ P40189-3^3^	NKRDLIKKHIWPNVPDPSKSHIAQWSPHTPPRHNFNSKDQMYSDGNFTDVSVVEIEANDKKPFPEDLKSLDLFKKEKINTEGHSSGIGGSSCMSSSRPSISSSDENESSQNTSSTVQYSTVVHSGYRHQVPSVQVFSRSESTQPLLDSEERPEDLQLVDHVDGGDGILPRQQYFKQNCSQHESSPDISHFERSKQVSSVNEEDFVRLKQQISDHISQSCGSGQMKMFQEVSAADAFGPGTEGQVERFETVGMEAATDEGMPKSYLPQTVRQGGYMPQ	918^1^ 857^3^			277	641^1^ 580^3^
	**IL-12Rβ2**	1,3		Q99665-1^1^ Q99665-3^3^	**STHYFQQK**VFVLLAALRPQWCSREIPDPANSTCAKKYPIAEEKTQLPLDRLLIDWPTPEDPEPLVISEVLHQVTPVFRHPPCSNWPQREKGIQGHQASEKDMMHSASSPPPPRALQAESRQLVDLYKVLESRGSDPKPENPACPWTVLPAGDLPTHDGYLPSNIDDLPSHEAPLADSLEELEPQHISLSVFPSSSLHPLTFSCGDKLTLDQLKMRCDSLML	862^1^ 776^3^			221	642^1^ 556^3^
		2		Q99665-2	**STHYFQQK***RRHSCPWTGS*	659	650	659	18	642
	**IL-23R**	1	IL23R1	Q5VWK5-1	NRSFRTGIKRRILLLIPKWLYEDIPNMKNSNVVKMLQENSELMNNNSSEQVLYVDPMITEIKEIFIPEHKPTDYKKENTGPLETRDYPQNSLFDNTTVVYIPDLNTGYKPQISNFLPEGSHLSNNNEITSLTLKPPVDSLDSGNNPRLQKHPNFAFSVSSVNSLSNTIFLGELSLILNQGECSSPDIQNSVEEETTMLLENDSPSETIPEQTLLPDEFVSCLGIV**NEELPSINTYFPQNILESHFNRISLLEK**	629			253	377
		2, 5, 6,7	IL23R2F2^2^ IL23R2F3^5^ IL23R6 ^6^ IL23R5^7^	Q5VWK5-2^2^ Q5VWK5-5^5^ Q5VWK5-6^6^ Q5VWK5-7^7^	**NEELPSINTYFPQNILESHFNRISLLEK**	375^2^ 264^5^ 374^6^ 227^7^			28	348^2^ 237^5^ 347^6^ 200^7^
	**IL-27R**	1		Q6UWB1-1	SGRCYHLRHKVLPRWVWEKVPDPANSSSGQPHMEQVPEAQPLGDLPILEVEEMEPPPVMESSQPAQATAPLDSGYEKHFLPTPEELGLLGPPRPQVLA	636			98	539
	**IL-12Rβ1**	1	Long	P42701-1	**GLNRAARHLCPPLPTPCASSAIEFPGGKETWQWINPVDFQEEASLQEALVVEMSWDKGERTEPLEKTELPEGAPELALDTELSLEDGDRC**KAKM	662			94	569
		2	Short	P42701-2	**GLNRAARHLCPPLPTPCASSAIEFPGGKETWQWINPVDFQEEASLQEALVVEMSWDKGERTEPLEKTELPEGAPELALDTELSLEDGDRC***DR*	660	659	660	92	569
	**LEPR** (OBR)	B	13.2, OBRb	P48357-1	**SHQRMKKLFWEDVPNPKNCSWAQGLNFQK**PETFEHLFIKHTASVTCGPLLLEPETISEDISVDTSWKNKDEMMPTTVVSLLSTTDLEKGSVCISDQFNSVNFSEAEGTEVTYEDESQRQPFVKYATLISNSKPSETGEEQGLINSSVTKCFSSKNSPLKDSFSNSSWEIEAQAFFILSDQHPNIISPHLTFSEGLDELLKLEGNFPEENNDKKSIYYLGVTSIKKRESGVLLTDKSRVSCPFPAPCLFTDIRVLQDSCSHFVENNINLGTSSKKTFASYMPQFQTCSTQTHKIMENKMCDLTV	1165			303	863
		A	6.4, HuB219.3	P48357-2	**SHQRMKKLFWEDVPNPKNCSWAQGLNFQK***RTDIL*	896	892	896	34	863
		C	12.1 OBRa	P48357-3	**SHQRMKKLFWEDVPNPKNCSWAQGLNFQK***MLEGSMFVKSHHHSLISSTQGHKHCGRPQGPLHRKTRDLCSLVYLLTLPPLLSYDPAKSPSVRNTQE*	958	892	958	96	863
		D	Hub219.2	P48357-4	**SHQRMKKLFWEDVPNPKNCSWAQGLNFQK***KMPGTKELLGGGWLT*	906	892	906	44	863
GROUP 3 – SOLUBLE ALFA CHAINS	**IL-6Rα**	1	Long	P08887-1	RFKKTWKLRALKEGKTSMHPPYSLGQLVPERPRPTPVLVPLISPPVSPSSLGSDNTSSHNRPDARDPRSPYDISNTDYFFPR	468			82	387
	**IL-11Rα**	HCR1	Membrane Form	Q14626-1	**W**LRLRRGGKDGSPKPGFLASVIPVDRRPGAPNL	422			33	390
		HCR2	Soluble Form, sIL11RA	Q14626-2	**W**	390			1	390
GROUP 4 – SLONG CHAIN RECEPTOR CHAINS	**IL-7Rα**	1	H20	P16871-1	**WKKRIKPIVWPSLPDHKKTLEHLCKKPRK**NLNVSFNPESFLDCQIHRVDDIQARDEVEGFLQDTFPQQLEESEKQRLGGDVQSPNCPSEDVVITPESFGRDSSLTCLAGNVSACDAPILSSSRSLDCRESGKNGPHVYQDLLLSLGTTNSTLPPPFSLQSGILTLNPVAQGQPILTSLGSNQEEAYVTMSSFYQNQ	459			196	264
		3	H1	P16871-2	**WKKRIKPIVWPSLPDHKKTLEHLCKKPRK***VSVFGA*	298	293	298	35	264
	**IL-9Rα**	1,2		Q01113-1^1^ Q01113-2^2^	**KLSPR**VKRIFYQNVPSPAMFFQPLYSVHNGNFQTWMGAHGAGVLLSQDCAGTPQGALEPCVQEATALLTCGPARPWKSVALEEEQEGPGTRLPGNLSSEDVLPAGCTEWRVQTLAYLPQEDWAPTSLTRPAPPDSEGSRSSSSSSSSNNNNYCALGCYGGWHLSALPGNTQSSGPIPALACGLSCDHQGLETQQGVAWVLAGHCQRPGLHEDLQGMLLPSVLSKARSWTF	521^1^ 500^2^			230	292^1^ 271^2^
		3		Q01113-3	**KLSPR***LGWGPTGPVCC*	342	332	342	16	327
	**IL-2Rβ** (IL-15R_β_)	1		P14784	NCRNTGPWLKKVLKCNTPDPSKFFSQLSSEHGGDVQKWLSSPFPSSSFSPGGLAPEISPLEVLERDKVTQLLLQQDKVPEPASLSSNHSLTSCFTNQGYFFFHLPDALEIEACQVYFTYDPYSEEDPDEGVAGAPTGSSPQPLQPLSGEDDAYCTFPSRDDLLLFSPSLLGGPSPPSTAPGGSGAGEERMPPSLQERVPRDWDPQPLGPPTPGVPDLVDFQPPPELVLREAGEEVPDAGPREGVSFPWSRPPGQGEFRALNARLPLNTDAYLSLQELQGQDPTHLV	551			286	266
	**IL-21R**	1		Q9HBE5	KTHPLWRLWKKIWAVPSPERFFMPLYKGCSGDFKKWVGAPFTGSSLELGPWSPEVPSTLEVYSCHPPRSPAKRLQLTELQEPAELVESDGVPKPSFWPTAQNSGGSAYSEERDRPYGLVSIDTVTVLDAEGPCTWPCSCEDDGYPALDLDAGLEPSPGLEDPLLDAGTTVLSCGCVSAGSPGLGGPLGSLLDRLKPPLADGEDWAGGLPWGGRSPGGVSESEAGSPLAGLDMDTFDSGFVGSDCSSPVECDFTSPGDEGPPRSYLRQWVVIPPPLSSPGPQAS	538			283	256
	**IL-4Rα**	1,3		P24394-1^1^ P24394-3^3^	CYVSITKIKKEWWDQIPNPARSRLVAIIIQDAQGSQWEKRSRGQEPAKCPHWKNCLTKLLPCFLEHNMKRDEDPHKAAKEMPFQGSGKSAWCPVEISKTVLWPESISVVRCVELFEAPVECEEEEEVEEEKGSFCASPESSRDDFQEGREGIVARLTESLFLDLLGEENGGFCQQDMGESCLLPPSGSTSAHMPWDEFPSAGPKEAPPWGKEQPLHLEPSPPASPTQSPDNLTCTETPLVIAGNPAYRSFSNSLSQSPCPRELGPDPLLARHLEEVEPEMPCVPQLSEPTTVPQPEPETWEQILRRNVLQHGAAAAPVSAPTSGYQEFVHAVEQGGTQASAVVGLGPPGEAGYKAFSSLLASSAVSPEKCGFGASSGEEGYKPFQDLIPGCPGDPAPVPVPLFTFGLDREPPRSPQSSHLPSSSPEHLGLEPGEKVEDMPKPPLPQEQATDPLVDSLGSGIVYSALTCHLCGHLKQCHGQEDGGQTPVMASPCCGCCCGDRSSPPTTPLRAPDPSPGGVPLEASLCPASLAPSGISEKSKSSSSFHPAPGNAQSSSQTPKIVNFVSVGPTYMRVS	825^1^ 810^3^			575	251^1^ 236^3^
	**βc**	1,2	IL-3Rb	P32927-1^1^ P32927-2^2^	RFCGIYGYRLRRKWEEKIPNPSKSHLFQNGSAELWPPGSMSAFTSGSPPHQGPWGSRFPELEGVFPVGFGDSEVSPLTIEDPKHVCDPPSGPDTTPAASDLPTEQPPSPQPGPPAASHTPEKQASSFDFNGPYLGPPHSRSLPDILGQPEPPQEGGSQKSPPPGSLEYLCLPAGGQVQLVPLAQAMGPGQAVEVERRPSQGAAGSPSLESGGGPAPPALGPRVGGQDQKDSPVAIPMSSGDTEDPGVASGYVSSADLVFTPNSGASSVSLVPSLGLPSDQTPSLCPGLASGPPGAPGPVKSGFEGYVELPPIEGRSPRSPRNNPVPPEAKSPVLNPGERPADVSPTSPQPEGLLVLQQVGDYCFLPGLGPGPLSLRSKPSSPGPGPEIKNLDQAFQVKKPPGQAVPQVPVIQLFKALKQQDYLSLPPWEVNKPGEVC	897^1^ 903^2^			437	461^1^ 467^2^
GROUP 5 – SHORT TAIL RECEPTOR CHAINS	**CRLF2**	1,3	TLSP-R IL-XR	Q9HC73-1^1^ Q9HC73-3^3^	KLWRVKKFLIPSVPDPKSIFPGLFEIHQGNFQEWITDTQNVAHLHKMAGAEQESGPEEPLVVQLAKTEAESPRMLDPQTEEKEASGGSLQLPHQPLQGGDVVTIGGFTFVMNDRSYVAL	371^1^ 259^3^			119	253^1^ 141^3^
	**γc**	1,2	IL-2Rg	P31785-1^1^ P31785-2^2^	ERTMPRIPTLKNLEDLVTEYHGNFSAWSGVSKGLAESLQPDYSERLCLVSEIPPKGGALGEGPGASPCNQHSPYWAPPCYTLKPET	369^1^ 179^2^			86	284^1^ 94^2^
	**IL-13Rα1**	1	IL-13RA1	P78552-1	KRLKIIIFPPIPDPGKIFKEMFGDQNDDTLHWKKYDIYEKQTKEETDSVVLIENLKKASQ	427			60	368
	**IL-13Rα2**	1	IL-13RA2	Q14627-1	RKPNTYPKMIPEFFCDT	380			17	364
	**IL-5Rα**	1,5		Q01344-1^1^ Q01344-5^5^	**CKI**CHLWIKLFPPIPAPKSNIKDLFVTTNYEKAGSSETEIEVICYIEKPGVETLEDSVF	420^1^ 211^5^			59	362^1^ 153^5^
		4		Q01344-4	**CKI***KLGPVRRKLKSSVI*	378	265	378	17	362
	**GM-CSFRα**	1, 7, 8	GMRα	P15509-1^1^ P15509-7^7^ P15509-8^8^	**KRFLRIQRLFPPVPQIKDKLNDNHEVEDE**IIWEEFTPEEGKGYREEVLTVKEIT	400^1^ 434^7^ 267^8^			54	347^1^ 381^7^ 214^8^
		2		P15509-2	**KRFLRIQRLFPPVPQIKDKLNDNHEVEDE***MGPQRHHRCGWNLYPTPGPSPGSGSSPRLGSESSL*	410	376	410	64	347
	**IL-3Rα**	1 + 2	SP1^1^ SP2^2^	P26951-1^1^ P26951-2^2^	RRYLVMQRLFPRIPHMKDPIGDSFQNDKLVVWEAGKAGLEECLVTEVQVVQKT	378^1^ 300^2^			53	326^1^ 248^2^

**#Full names of receptors are:** *PRLR* prolactin receptor; *GHR* growth hormone receptor; *EPOR* erythropoietin receptor; *TPOR* thrombopoietin receptor; (alternative name: myeloproliferative leukemia protein, MPL); *LIFR* leukemia inhibitory factor receptor; *OSMR* oncostatin-M-specific receptor subunit beta (alternative name: IL-31 receptor subunit beta); *G-CSFR* granulocyte colony-stimulating factor receptor; IL-31Rα, interleukin-31 receptor subunit alpha (alternative name: GLMR, Gp130-like monocyte receptor); *IL-6Rβ* interleukin 6 receptor beta (alternative name: gp130, glycoprotein 130); *IL-12Rβ2* interleukin 12 receptor beta 2; *IL-23R* interleukin 23 receptor; *IL-27R* interleukin 27 receptor; *IL-12Rβ1* interleukin 12 receptor beta 1; *LEP-R* leptin receptor (alternative name OB-R, obesity receptor); *IL-6Rα* interleukin 6 receptor alpha; *IL-11Rα* interleukin 11 receptor alpha; *IL-7Rα* interleukin 7 receptor alpha; *IL-9Rα* interleukin 9 receptor alpha; *IL-2Rβ* interleukin 2 receptor subunit beta (alternative name; IL-15Rβ, interleukin-15 receptor subunit beta); *IL-21R* interleukin 21 receptor; *IL-4Rα* interleukin 4 receptor alpha; *βc* cytokine receptor common subunit beta; *CRLF2* cytokine receptor-like factor 2 (alternative name TSLPR, thymic stromal lymphopoietin protein receptor); *γc* cytokine receptor common subunit gamma (alternative name *IL-2Rγ* interleukin 2 receptor subunit gamma); *IL-13Rα1* interleukin 13 receptor alpha 1; *IL-13Rα2* interleukin 13 receptor alpha 2; *IL-5Rα* interleukin 5 receptor alpha; *GM-CSFRα* granulocyte-macrophage colony-stimulating factor receptor subunit alpha; *IL-3Rα* interleukin 3 receptor subunit alpha

^a^Residue numbering is with the signal peptide included

^b^Isoform 2: missing residues 24–124, but ICD is identical

^c^SF1b, Isoform 8 missing 1–71 ICD identical to Isoform 6

^d^Isoform 4: A24D & 25–46 missing, but ICD is identical

The characteristic compositional bias of IDPs makes it possible to predict the degree of disorder in proteins computationally [109]. Almost 10 years ago, computational predictions of disorder was done for five of the C1CRs [110], but disorder predictors have since improved in quality and reproducibility [111], and no study has examined the entire family in unison. We therefore predicted the disorder profiles of the longest ICD isoforms of all 29 C1CRs as well as the propensity of regions to undergo folding-upon-binding using the ANCHOR scores [112] (Fig. 2a and SI Fig. S1). From these predictions, we observed that the ICDs of the entire family have high scores (> 0.5) for disorder along their complete sequence and none were predicted to harbor folded domains. Furthermore, almost all receptors had lower disorder scores in the juxtamembrane 20–50 residues, a region overlapping with the JAK1/2/3;TYK2 binding sites. Along the chains, regions of lower disorder propensity were observed, which at the same time were paralleled with high ANCHOR scores. Such signatures suggest the region to be prone to folding-upon-binding and thus constitutes a potential binding site [112]. Indeed, the dip with the lowest disorder score in PRLR-LF-ICD occurred around residue 610, which corresponds to the region of tyrosine phosphorylation by JAK2 (Y580/Y614), and docking site for STAT5 (YLDP). Comparing the profiles across the group 1 C1CRs revealed a similar pattern of disorder along the first 150–200 residues, although the extent of each of the regions with higher/lower disorder vary (Fig. 2a). Similar group specific profiles of some similarity in the first half of the ICDs were seen for groups 2, 3, and 4, but not for group 5 (SI Fig. S1); an observation likely reflecting their shorter ICDs. Finally, we compared the disorder profiles for the five different isoforms of the PRLR-ICD (SI Fig. S2). Despite the change in sequence, all the ICD isoforms were predicted to be disordered and with almost identical disorder profiles. This is consistent with the general observation that sequence may change in a family of proteins while the disorder-order profile persists [110, 113].

In summary, the predicted disorder profiles support that the ICDs of all the C1CRs are disordered (Fig. 2b) and highlight common disorder profiles with a distribution of binding sites prone to folding-upon-binding.

### The ICDs of the C1CRs have compositional biases distinguishing them from other IDPs

**Fig. 3:**
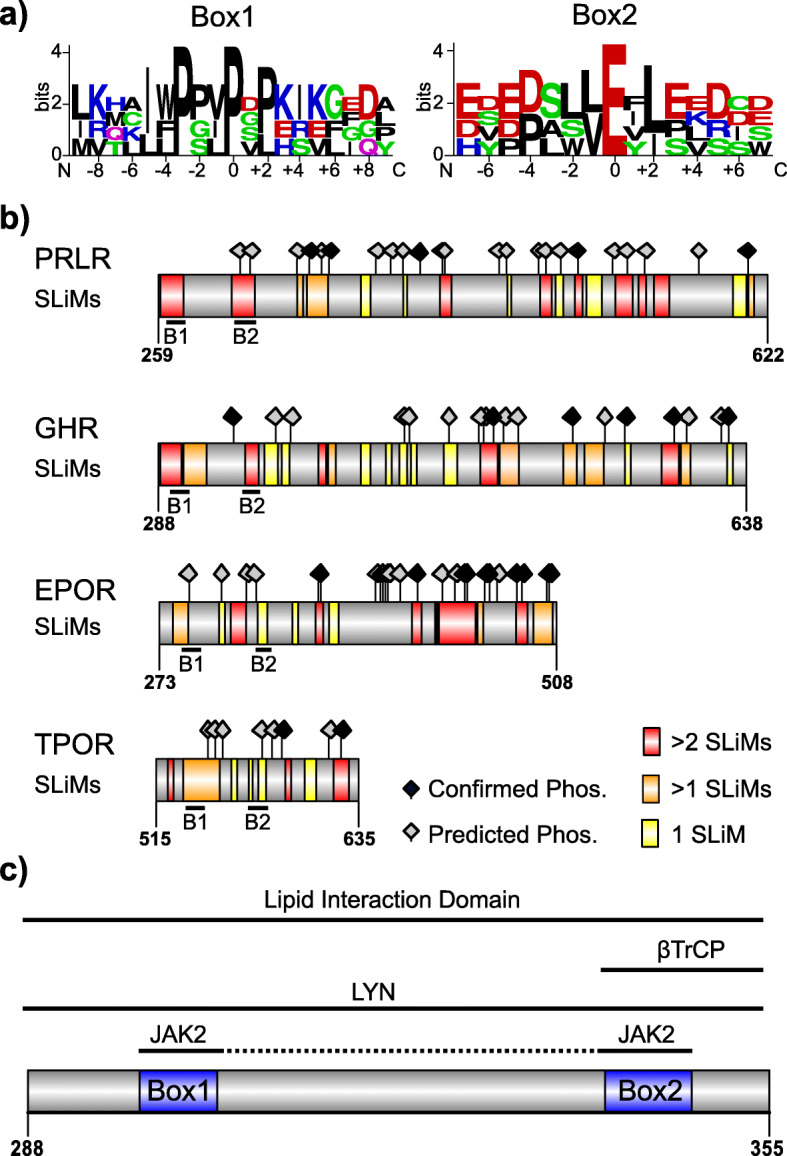
Short linear motifs form the basis for signaling choreography. **a** Sequence logo of the amino acid distribution in C1CR group 1 Box1 and Box2 motifs. Letter-height indicates the relative abundance of the given amino acid at the specific position. Red indicates an acidic residue, blue - a basic residue, green – a polar residue, black – a hydrophobic residue and pink – a neutral residue [102]. **b** SLiM and phosphorylation site mapping in C1CR group 1 ICDs. Regions with SLiMs are highlighted and colored according to the amount of overlap. Black indicates regions with no overlap. Yellow indicates regions with two overlapping SLiMs, whereas red indicates regions with more than two overlapping SLiMs. Predicted phosphorylation sites are indicated by grey diamonds [107] and confirmed and well-characterized sites by black diamonds. **c** Zoom on the membrane proximal 67-residue region of GHR-LF-ICD containing overlapping binding sites. Box1 and Box2 are highlighted in blue. Proteins were illustrated in IBS 1.0.3 [114]

To address if the C1CR-ICDs have physiochemical properties that distinguish them from other IDPs, we compared the amino acid content of the entire family (Fig. 2b) as well as the individual groups (SI Fig. S3) to those of folded proteins and other IDPs (for details see methods). The analysis revealed that the C1CR-ICDs indeed have global sequence compositions that stand out from other IDPs in three ways: First, some amino acids are depleted in the C1CR-ICDs, namely Met, Arg, Ala and Lys, which are less frequent than in general in IDPs and in folded proteins. Second, Cys, Trp, Leu and Val are significantly more frequent in the C1CR-ICDs than in other IDPs, and are as frequent as in folded proteins (except Val, which is less frequent than in folded proteins). Third, Pro is highly enriched in the C1CR-ICDs, and is even more frequent than in both folded proteins and IDPs in general. These differences are remarkable, but the role of these global compositional biases in C1CR functionality remains to be understood. The depletion in positively charged amino acids could be related to prevention of detrimental interactions with the negatively charged inner membrane leaflet to which the C1CR-ICDs are tethered through their TMDs, or with other negatively charged molecules. The enriched Cys, Trp, Leu, Val and Pro are in IDPs often found in SLiMs. Indeed, the saturation of SLiMs along the C1CR-ICD chains, as highlighted in Fig. 3 (see below), suggests enrichment in binding sites and may reflect large interactomes. Pro are known to preserve disorder in regions of IDPs with residual structural propensities [115], and hence could counter-balance the effects of the enrichment in hydrophobic residues. Furthermore, the chemistry of Pro causes rigidification of the backbone and consequently conformational expansion, as well as the formation of polyPro type II (PPII) structures by Pro-rich motifs [115]. Finally, several SLiMs and modification sites are Pro-based, including binding sites for JAKs and SH3s, and MAPK modification sites, which may increase the relative content of Pro in C1CR-ICDs.

Thus, even though C1CR-ICDs are classified as disordered from the disorder predictions, they have a remarkable compositional bias that distinguishes them from other IDPs, possibly due to SLiM enrichment.

Although sequence identity is often low among related IDPs, the sequence characteristics important for function are typically conserved, whether it being specific SLiMs, global conformational characteristics or specific functional domains [100]. Thus, regions of specific residue biases can be taken to represent domains of different chemical and structural properties, which may contribute differently to the function of the C1CR-ICDs. To identify putative functional domains of specific physio-chemical properties across the C1CR-ICDs we submitted the sequences to IDDomainSpotter [100]. IDDomainSpotter reveals distinct conformational biases in regions of long IDP sequences by calculating the fractions of specified residues in a sliding window of 15 residues, meaning, that for each residue k, the fraction of the specified residues between k-7 and and k + 7 is given. Here, we have analyzed e.g. the charge composition by setting Lys and Arg as positive contributions (+RK) and Glu and Asp (−DE) as negative contributors (net charge). Hence, a given residue k within the sliding window counts as + 1 if the position is a Lys or Arg, − 1 if the position is Glu or Asp, and 0 for any other residue.

The IDDomainSpotter analysis of the C1CR-ICDs revealed shared profiles for certain residues across the receptors (Fig. 2c), suggesting functional importance. First, they all shared a region of 10–20 residues in the region immediately following the TMD with a positive net charge, as typically observed for type I membrane proteins [30, 116]. This is followed by a region of ~ 50–60 residues (only ~ 20 residues for the shorter TPOR) with a negative net charge (Fig. 2c, green). We denominate these regions as the positive domain (PD) and the negative domain (ND), respectively. For the ICDs of PRLR and GHR, the PD has been shown to specifically interact with negatively charged lipids of the inner leaflet of the membrane [82]. However, the role of the ND is not understood. The negative charges may be relevant for membrane repulsion, or for compaction with the PD when not membrane bound. Alternatively, it could provide negatively charged flanking regions for specific SLiMs, such as for Box2 binding to SH2 domains or Pro-rich motifs binding to SH3 domains, as recently supported by experiments [117]. Second, ~ 100 residues and onwards from the TMD, the net charge was close to equally balanced along the chains. Another shared property is the almost equal distribution of the unusually abundant Cys and Pro throughout the chains (Fig. 2c, orange and purple). This could suggest that their abundance is related to global conformational properties, rather than e.g. interaction sites or PPIIs. The ICDs of group 1 further shared a pattern of depletion and enrichment (20–50 residues) of the hydrophobic branched amino acids Ile, Leu and Val throughout their chain (Fig. 2c, red). Such hydrophobic side chains are usually less abundant in IDPs because of the energetic penalty of solvation, and hence in IDPs they are often primarily located in e.g. SLiMs, or related to maintaining extended β-structures of relevance to binding. Finally, clustering of Phe, Tyr and Gly (+FYG) was analyzed, as IDRs enriched in these residues may be involved in liquid-liquid phase separation [118, 119], but no major clustering of these were present throughout the chains.

The patterns observed for the group 1 receptors is overall shared across the C1CR-ICDs, with the exception of some noteworthy variations in charge composition. Generally, all the C1CR-ICDs harbor a PD (sometimes in a shorter version), followed by an ND of various length, with subsequent close to net neutral charge along the chains. However, the short ICDs of group 3 only harbor a PD, and lack an ND. Furthermore, the ICDs of IL-31Rβ (OSMR) (group 2) and IL-4Rα (group 4) lack regions of substantial net charge throughout their ICDs, including the PD; a trait that may be related to their association with the IL-31Rα and IL-13Rα1, respectively.

### Short linear motifs allow expansion of the interactome

In the disorder profiles and the IDDomainSpotter analysis we observed distinct patterns, which may suggest the presence of multiple binding sites [120] (Fig. 2). For all receptors, the first region of low disorder propensity corresponds to the juxtamembrane region containing the most conserved and well-known motifs, Box1 and Box2, involved in JAK/TYK binding. As a Pro-rich motif, Box1 represents one of the most abundant SLiMs in the eukaryotic proteome [121]. The polyPro scaffold inherently provides a conformational bias towards PPII formation [122], which creates a structural predisposition that may drive an interaction via reduced entropic penalty of complex formation [123]. Except for group 5, which lacks canonical Box1 and Box2 motifs, all receptors harbor the conserved PXP motif in Box1 (SI Fig. S4), known to interact with the JAK-FERM domain [44, 124]. However, in most receptors, Box1 is further extended to PXXPXP (consensus for group 1: φφPXφPXP, where φ is any hydrophobic residue), which thereby accommodates both the minimal SH3 binding motif (PXXP) [125] and the FERM binding motif (PXP) in one combined SLiM (Table 2). This enables competitive binding to Src and JAK kinases [48, 126]. Although Box2 is remarkedly less conserved than Box1, sequence alignments reveal glutamates to be most abundant in Box2 (Fig. 3a). This is in accordance with their essential role as phospho-mimics in binding to the atypical JAK-SH2 domain [44–46]. Studies suggest JAK kinase binding to be driven by Box1 association to the FERM domain [45], which thereby increases the local concentration of Box2 to the SH2 domains, further suggesting Box1 as the primary anchoring point.

**Table 2:** Properties of disorder in C1CRs

IDP characteristic	Key descriptors for C1CR ICDs
Specific amino acid composition	The C1CR-ICDs have a unique amino acid composition that distinguishes them from both folded proteins and IDPs. *Depleted* in Met, Arg, Ala, and Lys compared to folded proteins and IDPs *Enriched* in Cys, Trp, Leu and Val compared to IDPs *Highly enriched* in Pro compared to folded proteins and IDPs
Disordered	All the ICDs of the C1CRs are predicted disordered throughout their sequences, but has been shown experimentally only for the GHR and PRLR
Rich in SLiMs	#Several SLiMs are common to groups of the C1CRs, in particular • BOX1 motifs (JAK/SH2) ΦΦP.ΦP.P (JAK2) ΦP. P (JAK1) ΦΦP.ΦP.[P/Φ].[P/Φ](JAK3/TYK2) • 14–3-3 R[^DE](0,2)[^DEPG][ST][^PRIKGN]-P R[^DE](0,2)[^DEPG][ST][^P]* • SOCS2/3 pY [AFILVWY].[AFILVWY] (loose SH2-motif) • PDZ ..[ST].[ACVILF]* (Class 1) ..[VLIFY].[ACVILF]* (Class 2) ..[DE].[ACVILF]* (Class 3) • TRAF2/6 [PSAT].[QE] E (TRAF2) [P].[Q]..D (TRAF2) [P].[Q]..[FYWHDE] (TRAF6) • STAT [Y]..[P] (STAT1) [Y]..[Q] (STAT3) [Y][VLTFIC].. (STAT5) (promiscuous) [Y]..[F] (STAT6) • Phospho-degrons D [S]G.(2,3[ST] [LIVMP].(0,2)(T)P..[ST] • Dileucine motifs [D/E]...[L/I][L/I] [D/E]..LL • Tyrosine-based internalization motifs Y..[LMVIF] Only very few SLiMs have been addressed experimentally and only 7 three-dimensional structures exist of complexes.
Rich in PTMs	All C1CR-ICDs have numerous predicted phosphorylation sites distributed along the chain, but only few have been confirmed by MS or by mutational studies. Some SLiMs are regulated by phosphorylation
Alternative splice variants	Out of a total of 29 C1CRs, 16 have at least two isoforms differing in their ICDs, but up to five ICD isoforms are seen for some receptors (PRLR, IL-31R). Isoforms allow for network rewiring by insertion and deletion of specific SLiMs
Dynamic conformational ensemble	The CIDER analysis and measured *Rg* of presentative C1CR-ICDs suggest that they take on a slightly compacted, but dynamic ensemble
Multispecificity	Overlapping SLiMs dominates C1CR-ICDs and allow competition as a regulatory mechanism. This is made possible as the disordered chain can adapts to several different binding partner.

#:^means that it cannot be a residue of this kind; * indicates the negatively charged C-terminal; For the JAK binding motifs, the similar PXP motif is shaded in grey. Φ illustrate ant hydrophobic residue

In the membrane distal region, SLiMs constituting docking sites for various signaling proteins have been mapped experimentally. These SLiMs are predominantly activated by phosphorylation to recruit SH2-containing proteins, such as those in STAT and SOCS proteins [127, 128]. Each group of receptors is known to preferentially recruit a specific STAT for activation [129] and each group therefore contains a specific subset of phospho-tyrosine motifs. Hence, group 1 harbors the STAT5 consensus motif, pYXXL [130], whereas the group 2 harbors the STAT3 consensus motif pYXXQ [131]. In addition to distinct down-stream signaling related SLiMs, many of the experimentally known SLiMs are also related to endocytosis, trafficking and degradation. Some of these are frequent and are well-described motifs experimentally, such as the dileucine-motifs (i.e. [D/E] XXX [L/I[L/I]] and [D/E]XXLL) seen in LIFR [132] and IL-2Rβ [133]), and the tyrosine-based motifs (i.e. YXXϕ), promoting clathrin-dependent endocytosis and internalization, first identified in the TPOR (YRRL) and later in the G-CSFR [134, 135]. Additionally, phosphorylation dependent degrons (i.e. DSGXXS) [136–138], promoting ubiquitin-dependent proteasomal degradation are also well characterized in both GHR and PRLR [136–138]. The motifs are summarized in Table 2.

For the longest ICD-isoforms of group 1, we subsequently predicted SLiMs and phosphorylation sites using the Eukaryotic Linear Motifs server (ELM) [106] and the iGPS [107], respectively (see methods), and mapped these to the sequences, marking those already experimentally confirmed (Fig. 3b). We made three important observations. First, the predicted SLiMs as well as their flanking regions are rich in amino acids that promote extended structures, such as Pro, Val and Leu [139], in accordance with the structures adapted in the bound states [79, 140, 141], and the compositional analysis made above. Second, it was evident that clusters of overlapping SLiMs are frequent and distributed across the ICD sequences, interleaved with stretches depleted in SLiMs (Fig. 3b**)**. Clusters of overlapping SLiMs may be scaffolding hot spots where multiple binding events can take place in a controlled manner, largely determined by binding competition, i.e. affinity, concentration and PTMs. However, IDPs may also accommodate simultaneous binding of several partners and thereby orchestrate signaling by bringing relevant proteins into close proximity [142]. As evident from Table 2, similarities between the tyrosine-based motifs are pronounced. Consequently, STAT and SOCS binding motifs may overlap, but also with phosphatase binding sites, such as e.g. for SHP2 [143–145], as well as with tyrosine-based internalization motifs. Thus, regulation of signaling fate by discrimination and availability via compressed motifs appear widespread in C1CRs and is critically linked to properties of disorder. Until recently, C1CR-regions with overlapping motifs have exclusively been characterized in the membrane distal regions. However, accumulating evidence suggest that also the membrane proximal regions contain SLiM-clusters. In GHR, the membrane proximal ~ 60 residues region contains a LID with an unknown function [82], a ubiquitin dependent degron whereto the E3-ligase βTrCP docks and promote GHR downregulation [136], as well as JAK2 [51] and Src kinase (LYN) binding sites [48] (Fig. 3c). JAK2 and LYN are the primary kinases in GHR signaling, controlling the activation of JAK2/STAT5 and MAPK pathways, respectively [48]. Both are known to be constitutively associated with the receptor ICD [48, 146] and their relative activation of pathways can be perturbed by mutations in the ECD affecting TMD alignment [48]. However, the molecular details of how the change in TMD alignment is associated with pathway selectivity are still unknown, but may be controlled by competitive binding of JAK2 and LYN and even further affected by membrane interaction. Similarly, GHR downregulation by βTrCP may likewise be driven by competition in binding. Thus, this region represents one of the essential composite SLiM-clusters in GHR with hitherto unexplored implications for the regulation of GHR signaling.

Typically, multiple binding events in IDPs are regulated by phosphorylations and can be characterized as binary on/off switches. However, accumulating evidence have revealed that phosphorylations can generate much more complex responses (reviewed in [147]), and multisite phosphorylation can additionally generate sensitive threshold responses as well as graded responses. The third important observation we made was that in the group 1 C1CRs, several phosphorylation sites were predicted, but only a small subset of these were well-characterized experimentally. Hence, much remains to be understood in terms of regulation and the many modification sites open the possibility that the C1CR-ICDs can have rheostat regulatory potential [17]. In this way, successive phosphorylations may additively increase (or decrease) binding affinity enabling graded responses, or they may modulate the conformational ensemble, with impact on signaling output. Importantly, multisite phosphorylations, which are functionally relevant for IDPs, remains to be addressed in the C1CRs.

In summary, interactions by C1CR-ICDs are primarily mediated by SLiMs, creating docking and modification sites for several accessory signaling proteins. Furthermore, clusters of overlapping SLiMs dominate the C1CR-ICDs, which together with the structural plasticity provided by the disorder properties, impart a unique condensed and versatile signaling scaffold, enabling establishment of large interactomes, whose content is controlled by the available pool and concentrations of interaction partners as well as PTMs. The spatio-temporal orchestration of signaling therefore rely on availability of the binding partner, affinities and kinetics and altogether eventually determine signaling fate.

### Network rewiring by isoforms

In order to investigate different C1CR-ICD isoforms at the molecular level, we took two approaches. First, we compared a long and a short isoform experimentally, using the PRLR as model, and second, we predicted and compared the presence of SLiMs for those C1CRs, which have longer isoforms that differ in the ICD sequence (i.e. having isoforms of unique sequence, not just truncations).

**Fig. 4:**
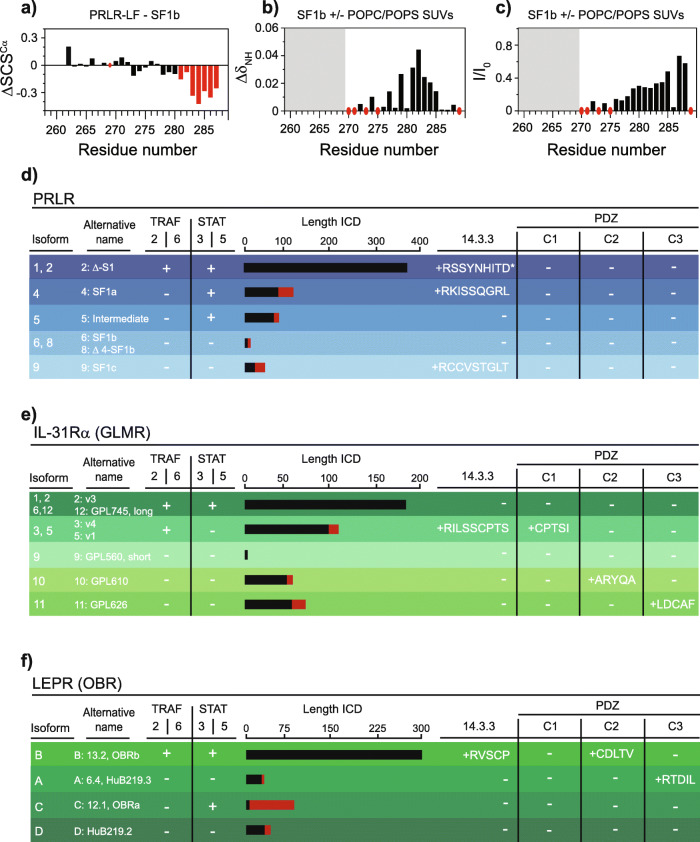
Network rewiring by disorder. Top; Comparison of a long and short isoform of PRLR. **a** Structural propensities of the PRLR-LF-ICD compared to PRLR-SF1b-ICD illustrated by differences in NMR secondary chemical shifts (SCSs) of C^α^. Red coloring indicates lower C^α^ SCSs in PRLR-SF1b-ICD compared to PRLR-LF-ICD, and hence less helicity. **b** NMR chemical shift perturbations upon addition of POPC/POPS SUVs to PRLR-SF1b-ICD and **c** intensity ratios of PRLR-SF1b-ICD in the absence and the presence of POPC/POPS SUVs. Red circles highlight prolines and/or unassigned residues and shaded areas loss of NMR signal upon addition. Bottom; Predicted and selected binding SLiMs in the isoforms of **d** PRLR, **e** IL-31Rα (GLMR), and **f** LEPR (OBR). Introduction of potential new binding SLiMs in the unique sequences are indicated by the sequence of the SLiM, and the presence of SLiMs in the LF of the isoforms is indicated with a “+”. When a SLiM is not present, this is indicated by a “-”. The length of the ICD is indicated by the scale bars on top, and red lines illustrate the length of the unique sequence. “*” indicates a different SLiM compared to the binding site identified by mutations in [148]. Alternative names for the isoforms are given in the second row. For other C1CR isoforms, see Table S2

First, we expressed and biophysically characterized PRLR-SF1b (residues 259–288) and compared it to PRLR-LF-ICD. PRLR-SF1b-ICD is much shorter (32 versus 386 residues) and differs only in the last three C-terminal residues, where K286, G287 and K288 of PRLR-LF-ICD are substituted by V286, T287, and P288 (P288 is the new C-terminus). Apart from the loss of multiple interaction sites by being shorter, including the loss of Box2, the chemical change from net positively charged to uncharged with more hydrophobic residues may influence the structural preferences as well as the interactome, especially membrane binding. From detailed NMR analyses, the PRLR-SF1b-ICD maintained structural disorder and dynamics (SI Fig. 5a-c), but transient helix 1, observed in the PRLR-LF-ICD, was eliminated in the PRLR-SF1b-ICD as seen by smaller secondary chemical shifts (SCSs) of C^α^ nuclei (Fig. 4a and SI Fig. 5c-d). This demonstrates that two isoforms differing only in three residues, may have different structural propensities (Fig. 4a). Functionally, however, and despite structural and chemical changes, membrane interaction using small-unilamellar vesicles of POPC:POPS (3:1) as membrane mimetics, previously observed for PRLR-LF-ICD [82], was preserved in PRLR-SF1b-ICD with loss of NMR signal intensities and chemical shift perturbations (Fig. 4b,c). Box1 was previously suggested to interact with cyclophilin A (CypA) [149], and Box1 proline *cis/trans* isomerization claimed important for this interaction [84]. However, form NMR chemical shift analyses (SI Table S1), Pro *cis/trans* isomerization appeared not to be dominant in the free state of neither isoforms, and accordingly none of them interacted with CypA (SI Fig. 5f-i). Thus, despite sequence and structure differences between the two isoforms, functionality was maintained in the short isoform. In this case, shortening of the ICD by removing 90% of it, including numerous SLiMs and phosphorylation sites, as well as docking sites for STAT and SOCS, in this case only resulted in a major reduction in the interactome.

Shorter isoforms as described for PRLR exist for 9 of the C1CRs. However, many ICD isoforms have longer regions of unique sequence, which differ from the canonical isoform. Thus, to explore if these longer ICD isoforms may have gained new interactions sites, we predicted which common SLiMs were gained or lost, disregarding potential phosphorylation sites and receptor-unique SLiMs **(**Table S2). The unique sequences were found to carry distinct SLiMs. In the case of group 1, a 14–3-3 binding SLiM has previously been identified in PRLR isoform 1 [148]; a SLiM originally discovered active in cytokine receptors in the IL-9R [150]. However, predictions suggested the presence of a different 14–3-3 motif, just six residues C-terminal to the experimentally described site (Fig. 4d**,** SI Table S2). Compared to the PRLR-LF-ICD, the short forms did not possess the STAT-docking sites or the 14–3-3 binding SLiM. Instead, a different 14–3-3 binding SLiM each with different sequence properties was predicted in the unique sequences (SF1a and SF1c) (Fig. 4d). For TPOR, isoform 2 had two 14–3-3 SLiMs, which were absent in isoform 1, while for the GHR and EPOR, which do not have any known 14–3-3 binding SLiMs, the isoforms with unique sequences (isoform 2 and 3 of GHR and isoform T of EPOR) were much shorter, and without any predictable common or different SLiMs. Thus, for PRLR, the preservation of the 14–3-3 SLiM despite changes in sequence suggest a key regulatory function, one of which may be to attenuate receptor signaling as suggested [148]. Relevantly, the LFs of EPOR, GHR and PRLR all had a phosphorylation dependent degron, interacting with the SkpSCF-betaTrCP1 complex or the Skp1_Cullin-Fbox, leading to ubiquitylation and degradation, as shown experimentally for PRLR and GHR, where it negatively regulates receptor stability [136, 137]. These were not identified in any of the shorter isoforms, which also have been seen to be stabilized on the membrane [151]; a possible result of the lack of associated proliferative signaling and hence lack of need for immediate down regulation.

For other receptors, e.g. the IL-31Rα (GLMR) and LEPR isoforms, we found that unique sequences introduced PDZ binding SLiMs at the new C-termini (Fig. 4e). Furthermore, when the PDZ motif is present, each IL-31Rα (GLMR) receptor isoform had a unique PDZ-binding SLiM (expect for isoforms 3 and 5, which are identical in their ICDs), allocating the isoforms to interact with different classes (Class 1,2 and 3) of PDZ domains (Fig. 4e) [152, 153]. The same was true for the LEPR, where each isoform has a unique PDZ binding SLiM (Fig. 4f). In fact, the introduction of a PDZ SLiM in the C-terminus in one isoform was observed for several receptors including G-CSFR, IL-7Rα, IL-9Rα, and GM-CSFRα (Table S2). Why these isoforms need PDZ binding motifs is not clear, but several scaffolding proteins with specialized subcellular localization and tissue specificity exist, known to contain multiple PDZ domains by which they orchestrate supramolecular complexes. Binding of the IL-5Rα-ICD to a PDZ domain from syntenin (Fig. 1 b) [81] supports the involvement of further scaffolding proteins for formation of larger signaling complexes. PDZ containing protein may be of relevance to the C1CRs and could engage proteins from the NHERF and PSD-95 families [154], which also scaffold kinases as Fyn [155]. Alternatively, E3-ligases belonging to the MARCHs family coordinate binding via PDZ domains and are relevant for ubiquitylation of proteins in the intracellular membranes [156]. However, besides the complex between the IL-5Rα-ICD and the PDZ domain from syntenin, complexes of C1CRs with PDZ domains remain to be experimentally explored. Finally, for all receptors with isoforms, the longest isoform, except for GM-SCFRα, carries the interaction with STATs, either STAT5 or STAT3 or both, but, additionally also carry a binding motif for TNF receptor-associated factor (TRAF)-2 or TRAF-6, none of which are found in other, shorter isoforms. In a few cases, the STAT and/or TRAF motifs are maintained in the second longest isoform, and sometimes a shift between STAT5 and STAT3 or between TRAF-2 and TRAF-6 occurs.

Thus, for the C1CRs, the disorder predictions and experimental characterization of selected representatives have suggested that the isoforms maintain structural disorder, and their presence suggests several mechanisms by which disorder orchestrates signaling. The first is the complete removal of a large part of the ICD, eliminating SLiMs important for STAT activation, TRAF interaction and downregulation by degradation via degron activation. In this way the shorter isoforms act as negative regulators, or decoy receptors, of signaling, as seen for the short forms of the PRLR and GHR [62, 151]. However, these isoforms still maintain binding capacity as seen from for membrane binding of PRLR-SF1b above. The second mechanism by which isoforms orchestrate signaling is via rewiring of the interactome to access completely new networks, exemplified by the addition and removal of binding sites for e.g. 14–3-3 proteins and PDZ domains. This allows for different signaling profiles dependent on expression profiles of the C1CR-ICD isoforms. However, more studies into network rewiring of the C1CRs are warranted, and the analysis made here merely provides a starting point.

### The conformational ensembles of C1CR-ICDs

IDPs are functional without taking on a single, well-defined tertiary structure. Yet, they cannot adequately be described as simple statistical coil chains equally populating all possible conformations allowed by their backbone torsion angles. Instead, IDPs display varying degrees of compaction and elongations, and contain transient, short- and long-range structural organizations. Hence, the disorder of the C1CR-ICDs not only infer flexibility and high accessibility of binding sites, but certain chain dimensions and spatial organizations may influence the organization of the signaling complexes and orchestration of protein interactions, and in the end, signaling outcome. Currently, the conformation and dimensions of IDPs cannot be quantitatively predicted from sequence [157, 158]. Nonetheless, the balance between chain-chain and chain-solvent interactions that determines the conformational preference is related to specific sequence features that influence the conformational ensembles in predictable ways [101, 157, 159, 160]. One set of these relates to global compositional sequence features (i.e. parameters that are independent on the sequence order), and the fraction of charged residues and the net charge per residue are particularly important [159, 161]. In addition, features relating to sequence patterning, especially the patterning of oppositely charged residues and expansion promoting residues, influence compaction [158]. However, the current difficulties in consistently predicting the conformational ensemble of all IDPs reflects that some of these behaviors are encoded in sequence features yet to be unraveled.

IDPs have been classified into five compositional groups in a diagram of states [161] based on their fraction of positively charged residues (f^+^) and fraction of negatively charged residues (f^−^). These two global parameters are combined into two measures underlying a diagram of states: the fraction of charged residues (FCR = f^+^ + f^−^) and the net charge per residue (NCPR = f^+^ − f^−^). An explanation of the relation between these parameters and the properties of the chain is given in the supplemental data. Of 879 IDRs longer than 15 residues found in DisProt, CIDER [101] classified 40% as belonging to R1, 35% to R2, 22% to R3, and 3% to either R4 or R5 [157]. For each C1CR-ICD, the sequence of isoform 1 was submitted to CIDER [101], except for LEPR and G-CSFR, for which isoform B and 3, respectively, were selected as these were the longest isoforms (see Table 1). For GM-SCFRα, both isoform 1 and 2 were analyzed because they differed in more than 50% of their C-terminal sequences (see Table 1). The C1CR-ICDs generally fell close to the boundary between R1 and R2, with most belonging to R1 (61%) (Fig. 2a), suggesting a preference for compact, but still dynamic, heterogenous conformational ensembles [157]. Nonetheless, in particular for sequences belonging to R2, their overall charge neutrality means that their conformational preference cannot be predicted from global composition alone [157, 159]. Furthermore, it should be noted that the boundary between R1 and R2 has been determined ad hoc, and has been suggested to be positioned at lower FCR for longer sequences [157, 159]. Furthermore, for ICDs > 100 residues or with a high proline fraction (> 0.15), no qualitative prediction of the conformation can be made for sequences of R1, as these tend to have more extended conformations than their scores predict.

Since almost all the C1CR-ICDs are long IDPs of R1 and R2, the conformational preferences cannot be predicted from global composition alone but may also be influenced by e.g. sequence patterning. Particular the patterning, or mixing, of oppositely charged residues is important, as well as expansion driving- and aromatic residues. The parameter κ reports on how well positively and negatively charged residues are segregated across the sequence and is normalized between 0 and 1, with κ close to zero representing sequences with evenly distributed charges, while sequences with κ close to 1 have highly segregated charges. It has been shown that as κ approaches 1, the conformational ensemble becomes more compact [161]. However, since κ is calculated by normalizing to the most segregated sequence within the given composition, a specific κ value will not have the same meaning for two sequences with different FCR and |NCPR| values. Furthermore, for long IDRs, such as most of the C1CR-ICDs, κ is calculated only within a window of 5 and 6 residues, ignoring long-range effects. κ is most informative for sequences with an FCR above 0.25 and NCPR between − 0.1 and + 0.1, for which a κ below 0.12 is considered low and a κ above 0.25 is high. Especially for polyampholytic sequences with an FCR beyond 0.4, charge patterning is predicted to have a major impact on the conformation [160]. There is one such example, namely the GM-CSFRα, which has an FCR of 0.41, an NCPR of − 0.07 and a κ of 0.25, suggesting chain compaction.

The position of the far majority (94%) of the C1CR-ICDs in R1 and R2 is a consequence of their low net charges. Their FCR values are in the mediocre range of 0.1 < FCR < 0.3 [160], while at the same time, their NCPR is close to 0, demonstrating that they are near-symmetrical polyampholytes. For the C1CR groups with long ICDs (1, 2 and 4), the group average FCRs (0.21; 0.23; 0.19) and NCPRs (− 0.06; − 0.06; − 0.05) are remarkably similar, suggesting that charge properties are a conserved trait. The similarity of these parameters also allows us to compare their κ values more directly, going from a group average of 0.20 for group 1, 0.18 for group 2 to 0.22 for group 4. This is consistent with the IDDomainSpotter analysis presented earlier (Fig. 2c). Here we found that almost all of the C1CR-ICDs harbored a PD immediately following their TMD, succeeded by an ND, and with net charge neutral regions for the remainder of the chain. Together, this suggest that the influence of the global charges and the charge patterning on the conformational ensembles are consistent throughout group 1, 2 and 4, except for IL-31Rβ (OSMR) (group 2) and IL-4Rα (group 4). As mentioned, the shorter ICDs of group 3 and 5 result in somewhat different global charge properties.

**Fig. 5:**
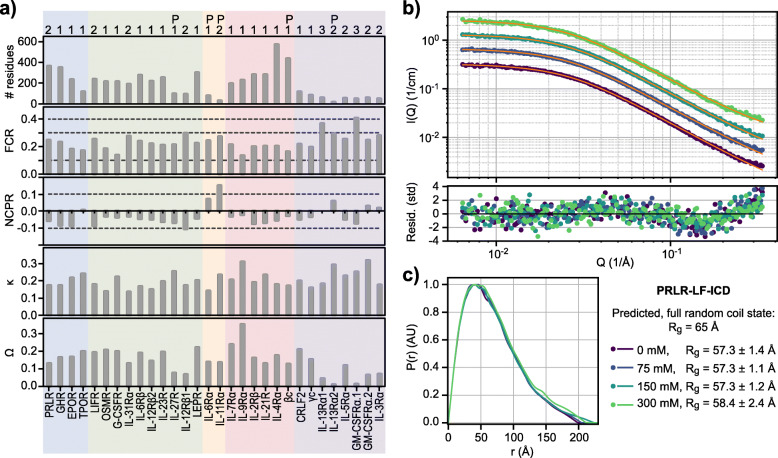
Conformational properties of C1CR-ICDs. **a** Plot of conformational parameters provided by CIDER analyses of the longest isoforms of the C1CR-ICDs. For GM-SCFRα, both isoform 1 and 2 were analyzed as they differed > 50%. Each receptor group is highlighted with different colors; 1 – blue, 2 – green, 3 -yellow, 4 – red, and 5 - purple. When sequences have a Pro warning, i.e. a Pro fraction > 0.15, the ICD is highlighted with a P on top. The numbers at the top are the compositional region assigned by CIDER. # residues are the numbers of residues in the ICD, FCR is the fraction of charged residues (FCR = f^+^ + f^−^), NCPR is the net charge per residue (NCPR = f^+^ − f^−^), κ is a measure of the charge mixing across the sequence (normalized between 0 and 1) and Ω additionally takes Pro mixing into account (normalized between 0 and 1). The dotted lines at 0.4, 0.3, and 0.1 in the FCR plot represent the borders for very highly charged (above), highly charged (above) and highly depleted (lower) in charges, respectively [160]. The area between the dotted lines at 0.1 and − 0.1 in the NCPR plot represents the range occupied by 70% of human IDPs [160]. **b** Experimental SAXS data (spheres) and Gaussian random coil model fit (lines) for PRLR-LF-ICD at different concentrations of NaCl (see color key), with the residuals from the fit shown below in units of standard deviations. **c** Pair distance distribution functions for the PRLR-LF-ICD SAXS data. The color coding is the same as in b)

The Ω parameter both describes the patterning of the charged residues as well as of proline. Like for κ, Ω is normalized between 0 and 1, with Ω close to zero representing sequences with evenly distributed charges and prolines, while sequences with Ω close to 1 have highly segregated charges and prolines [162]. It has been shown that when Ω approaches 1, the preference for expanded conformations increases [162]. A high fraction of Pro (> ~ 15%) may cause more expanded conformations as Pro prefers to be solvated and promotes stiffness. Five of the C1CR-ICDs had a high fraction of Pro (15–19%): IL-27R, IL-6Rα, IL-11Rα, βc, IL-13Rα2 (Fig. 5a**, top**). The amino acid fraction and IDDomainSpotter analysis (Fig. 2) revealed that the Pros are unusually abundant in the C1CR-ICDs and close to equally distributed. From the CIDER analysis we found that Ω, like κ, is similar for many of the C1CR-ICDs, but is lower for the shorter sequences (Fig. 5a). This could simply be a consequence of the Pro-rich Box1 sequences, leading to relatively higher proline scores in the shorter sequences.

To summarize, the theoretical analysis of the C1CR-ICD sequence parameters known to influence compaction suggest that many of them may be similarly biased towards a specific degree of extension or compaction of their conformational ensembles, but that this degree cannot be predicted from sequence. Hence, to determine this bias, we experimentally investigated the degree of compaction and its responsiveness to salt by SAXS using two long, representative C1CR-ICDs, namely that of PRLR-LF-ICD (Fig. 5b,c) and GHR-LF-ICD (SI Fig. S6). The SAXS profiles of the PRLR-LF-ICD were consistent with those expected for fully disordered proteins, and was fitted to an *Rg* of 57.3 ± 1.4 Å in 20 mM phosphate buffer. The predicted *Rg* of the PRLR-LF-ICD for a fully random coil state is, according to Kohn et al. [98], 65 Å, suggesting that the PRLR-LF-ICD populate a slightly compacted ensemble. The pair distance distribution function (*P(r)*), which is a histogram of distance distributions within the protein, peaks at ~ 45 Å and has a *D*_*ma*x_ of ~ 200 Å. Increasing the concentration of salt to 300 mM did not significantly affect the fitted *Rg* nor the *P(r)* distribution (Fig. 5b,c), suggesting that the global degree of compaction of PRLR-ICD is not sensitive to salt, as otherwise often observed for more charged IDPs [163], perhaps related to the high content of Pro and branched amino acids. The same trends were observed from SAXS on the GHR-LF-ICD, having a similarly slightly compacted ensemble that was insensitive to salt (SI Fig. S6). Hence, the ICDs across the C1CR family having similar global charge properties and patterning (Fig. 5a), may populate similarly compacted ensembles as also indicated from the Ω scores (0.15–0.35), although this remains to be experimentally more broadly verified.

### Versatile and controlled orchestration of signaling by unique structural disorder in C1CRs

It is remarkable that the entire family of C1CRs, differentiating into > 50 isoforms, are all predicted to be disordered in their entire ICD sequence. Nonetheless, the disordered ICDs are critically understudied, leaving us with a naive and too simplistic schematic view of the ICDs as passive strings of varying lengths with kinases constitutively attached. In the present paper we have highlighted the properties linked to disorder responsible for controlling the diverse signaling by C1CRs (Table 2) and asked: Why has disorder been selected for governing intracellular C1CR signaling? Their complete disordered nature stands in contrast to the majority of other types of single-pass transmembrane receptors such as the receptor tyrosine kinases, where intracellular signaling is mainly governed by intrinsic kinase activity. We have here shown that the long disordered ICDs of C1CRs are brimming with clusters of multifunctional SLiMs throughout their length, suggesting that one explanation is the signaling versatility and scaffolding capacity of this type of ICD. Furthermore, we have outlined that overlapping SLiMs are prevalent in the C1CR-ICDs, hinting that the disordered ICDs further allow for complex regulation of diverse signaling through competition and regulation of interactions with a plethora of different binding partners through multispecificity. Thereby, activation becomes dependent on the coupled equilibria and kinetics of two (or more) binding events. Indeed, the ability of a distinct region in the disordered ICD to bind to many different proteins is facilitated by structural adaptation and folding-upon-binding [164, 165]. Additionally, the C1CR-ICDs are hot spots for multiple phosphorylation events of which only a few are well-characterized as binary on-off switches. This directly – or indirectly – affect affinities and additionally expands the number of states accessible by the chain at any time. We have further suggested that an additional layer to this regulation is added by the existence of different C1CR-ICD isoforms, in which entire groups of SLiMs can be eliminated and new ones added, a feature that is much easier for IDPs to successfully obtain during evolution compared to folded proteins. By controlled expression of the isoforms, a complete rewiring of the interaction network can be done. Hence, the full disordered nature of the C1CR-ICDs allows for a fascinatingly versatile and complex interaction hub.

Can such signal complexity be facilitated through a simple string with kinases attached? Our sequence analysis and experimental studies have revealed biases in the C1CR-ICDs that differentiate them from being simple statistical coils. They have conserved distinct compositional biases that differentiate them from other IDPs; biases that are distributed throughout their chains, including the presence of disordered domains of specific physiochemical properties. This suggests that these compositional biases are representing shared functionalities yet to be characterized. Our experimental SAXS data on the long ICDs of the archetypical receptors GHR and PRLR revealed that they are slightly more compacted than expected for a fully random coil (~ 57 Å versus 65 Å for PRLR-LF-ICD). This indeed suggests an inherent conformational bias based on the conservation of certain sequence properties maintained across the family. Importantly, however, it should be kept in mind that in the cell, the C1CR-ICDs are most likely never completely void of interactions at any point. Previous characterizations of PRLR-LF-ICD and GHR-LF-ICD have revealed the presence of LIDs, further suggesting distinct organizational features at the membrane interface where also many kinases are tethered. Additionally, we found that the same sets of SLiMs are placed differently in the C1CR-ICDs with variable distances, which may provide an additional tuning of the signaling outcome, both via the length of their disordered spacers as well as the properties of these [166]. Thus, SLiM organization within the chain may imprint different affinities in different complexes despite their exploitation of identical SLiMs, providing an additional layer of the spatio-temporal orchestration of signaling. In fact, is it possible that disordered cytoplasmic domains generally can be classified by their collection of SLiMs, providing specific SLiM catalogues of disordered membranes proteins, but such decomposition will require a much broader analysis across many different protein families.

## Conclusion

In conclusion, we suggest that the C1CR-ICDs are far from simple strings with constitutively bound kinases. Rather, they carry both organizational and operational features left uncovered in their disorder, but of key importance for understanding orchestration of signaling. How these features operate in the higher-order oligomers of the C1CRs, bringing ICDs from several chains in close proximity, increases the dimension of future studies. For example, the mere volume taken up by more chains may allow them to generate higher order ensembles of specific properties. In such disordered reaction chambers, lower affinity interactions may be boosted and even shared between chains adding features, mechanisms and complexities to regulations, also yet to be discovered. Taken together, it is evident that the understanding of the fascinating ability of these long, completely disordered chains to orchestrate complex signaling pathways is still in its infancy.

## Supplementary information

**Additional file 1 Figure S1**: Disorder prediction for group 2, group 3, group 4 and group 5 C1CRs, but not the three common receptors. **Figure S2**: Disorder prediction for PRLR isoforms. **Figure S3**: Fractional differences in composition between the different C1CR-ICD groups or a set of IDPs, and a set of folded proteins calculated for each amino acid type. **Figure S4:** Sequence logos for Box1 shown for group 2, group 3, group 4 and group 5. **Figure S5**: R1 and R2 relaxation rates for PRLR-SF1b-ICD. **FigureS6**: Small-angle X-ray diffraction analyses of GHR-LF-ICD. **Table S1**: Proline *cis-trans* populations in PRLR-LF-ICD and PRLR-SF1b-ICD. **Table S2**: Overview of SLiMs lost and gained in C1CRs isoforms with unique sequences. Supplemental data: Interpretation of the diagram of states and conformational properties.

## Data Availability

All data generated or analyzed during this study are included in this published article and its additional information files.

## Abbreviations

C1CR: Class 1 cytokine receptors
CD: Circular dichroism
ECD: Extracellular domain
FCR: Fraction charged residues
ICD: Intracellular domain
IDP: Intrinsically disordered protein
LID: Lipid interaction domain
NCPR: Net charge per residue
NMR: Nuclear magnetic resonance
PPII: Polyproline II
PTM: Post-translational modification
SAXS: Small angle X-ray scattering
SCS: Secondary chemical shift
SLiM: Short linear motif
SUV: Small unilamellar vesicles
TMD: Transmembrane domains
