# Supplementary material for: Orchestration of signaling by structural disorder in class 1 cytokine receptors
Source: Cell Commun Signal. 2020 Aug 24;18:132. doi: 10.1186/s12964-020-00626-6 (PMC7444064; doi:10.1186/s12964-020-00626-6)
Supplement: Supplementary file 2 — Additional file 1 Figure S1: Disorder prediction for group 2, group 3, group 4 and group 5 C1CRs, but not the three common receptors. Figure S2: Disorder prediction for PRLR isoforms. Figure S3: Fractional differences in composition between the different C1CR-ICD groups or a set of IDPs, and a set of folded proteins calculated for each amino acid type. Figure S4: Sequence logos for Box1 shown for group 2, group 3, group 4 and group 5. Figure S5: R1 and R2 relaxation rates for PRLR-SF1b-ICD. FigureS6: Small-angle X-ray diffraction analyses of GHR-LF-ICD. Table S1: Proline cis-trans populations in PRLR-LF-ICD and PRLR-SF1b-ICD. Table S2: Overview of SLiMs lost and gained in C1CRs isoforms with unique sequences. Supplemental data: Interpretation of the diagram of states and conformational properties. [file 12964_2020_626_MOESM2_ESM.docx]

**SUPPLEMENTAL INFORMATION**

**Orchestration of signaling by structural disorder in class 1 cytokine receptors**

Pernille Seiffert^1,2#^, Katrine Bugge^1,2#^, Mads Nygaard^1,2^_,_ Gitte W. Haxholm^1,2^, Jacob H. Martinsen^1,2^, Martin N. Pedersen^3^, Lise Arleth^3^, Wouter Boomsma^4^, Birthe B. Kragelund^1,2,*^

^#^contributed equally

List of supplemental figures

**Fig. S1**: Disorder prediction for group 2, group 3, group 4 and group 5 C1CRs, but not the three common receptors

**Fig. S2**: Disorder prediction for PRLR isoforms

**Fig. S3**: Fractional differences in composition between the different C1CR-ICD groups or a set of IDPs, and a set of folded proteins calculated for each amino acid type

**Fig. S4**: Sequence logos for Box1 shown for group 2, group 3, group 4 and group 5

**Fig. S5**: **Comparison of molecular details of PRLR-ICD isoforms**

**Fig. S6**: Small-angle X-ray diffraction analyses of GHR-LF-ICD

List of supplemental tables

**Table S1**: Proline *cis-trans* populations in PRLR-LF-ICD and PRLR-SF1b-ICD

**Table S2**: Overview of SLiMs lost and gained in C1CRs isoforms with unique sequences

List of supplemental data:

Interpretation of the diagram of states and conformational properties

**Fig. S1. Disorder prediction for C1CRs.** **a)** group 2, **b)** group 3, **c)** group 4 and **d)** group 5. The disorder propensity, ranging from 0 to 1 was predicted using IUPred2A (blue), ANCHOR (black) and Pondr-fit VSL2 (red) (see methods) and is plotted as a function of residue number. The boundaries between the ECD (white background), TMD (yellow background) and ICD (orange background) were predicted using TMHMM v. 2.0 (see methods). The numbering of the sequences includes the signal peptide.

**Fig. S2: Disorder prediction for PRLR isoforms**. The isoform is indicated at the top of each plot. The disorder propensity, ranging from 0 to 1 was predicted using IUPred2A (blue), ANCHOR (black) and Pondr-fit VSL2 (red) (see materials and methods) and is plotted as a function of residue number. The boundaries between the ECD (white background), TMD (yellow background) and ICD (orange background) were predicted using TMHMM v. 2.0. The sequence numbering includes the signal peptide.


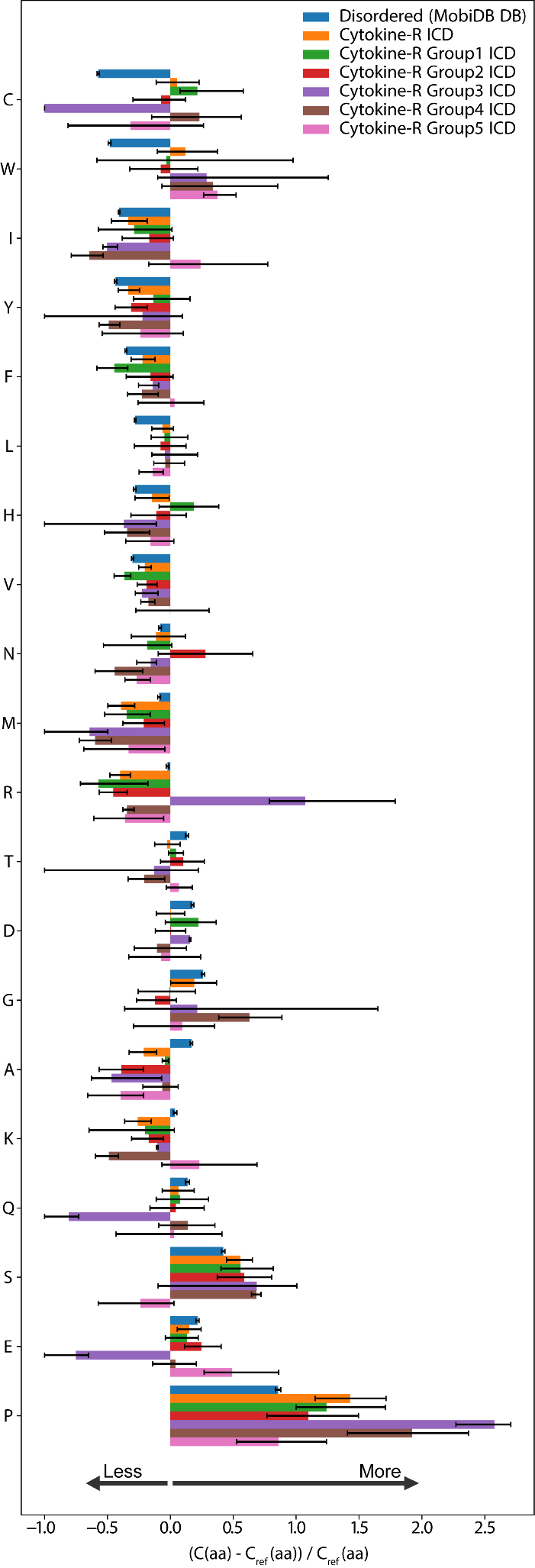
**Fig. S3: Amino acid distribution in C1CRs distributed pr. group**. The groups are colored according to the legends. The arrows indicate the directions of “more” abundant than in folded proteins, and “less” abundant than in folded proteins. The error bars indicate the 90% confidence interval of the estimated frequencies, calculated using a per-protein bootstrapping procedure with 1000 iterations [1]. Please note that some of the groups only consist of very few sequences (particularly group 3), and the bootstrap procedure is therefore associated with substantial uncertainty.

**Fig. S4. Sequence logos for Box1**. The sequences are from **a)** group 2, **b)** group 3, **c)** group 4 and **d)** group 5 and the loges were produced using Weblogo3.0 (see methods).


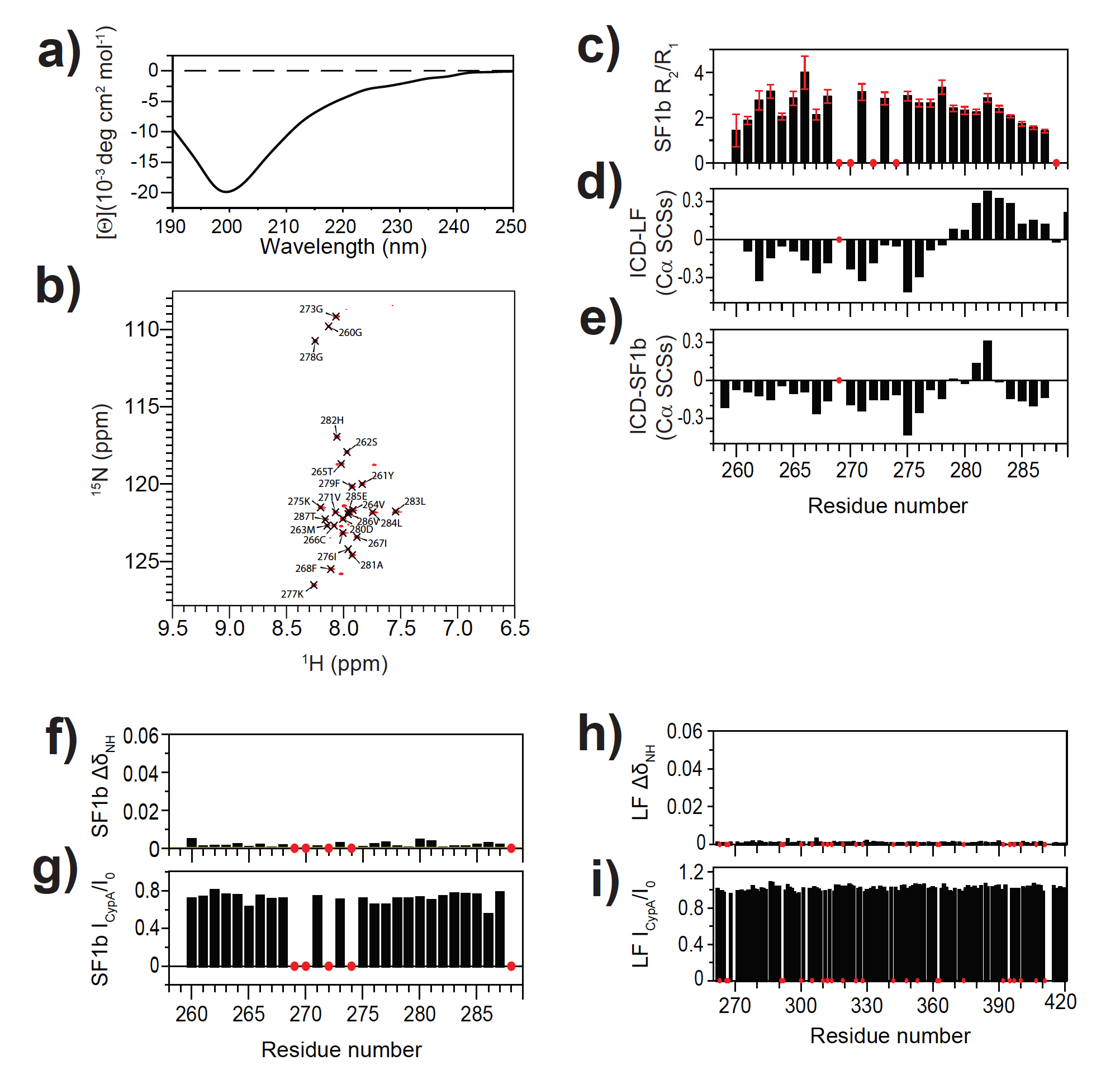


**Fig. S5. Comparison of molecular and functional details of two PRLR-ICD isoforms. a)** Far-UV CD spectrum and **b)** ^1^H,^15^N-HSQC spectrum of PRLR-SF1b-ICD**. c)** Ratios of *R_2_* and *R_1_* relaxation rates of PRLR-SF1b-ICD. Error bars represent errors from the fit, and red circles highlight prolines and/or unassigned residues. Longitudinal relaxation rates (*R*_1_) of the backbone amides with an overall similar value for all residues with an average of 1.30 ± 0.07 s^-1^ and transverse relaxation rates (*R*_2_) of backbone amides with an average value of 3.2 ± 0.3 s^-1^, with a plateau in the middle of the peptide for which the average *R_2_* rate was 3.6 ± 0.3 s^-1^. NMR secondary chemical shifts (SCSs) of C^α^ for **d**) PRLR-SF1b-ICD and **e**) PRLR-LF-ICD, positive values indicated transient helicity, negative values transient extended structures. Chemical shift differences of amides and intensity ratios in the absence and presence of CypA for **f,g**) PRLR-SF1b-ICD and **h,i**) PRLR-LF-ICD, respectively. Red circles highlight prolines and/or unassigned residues.

**
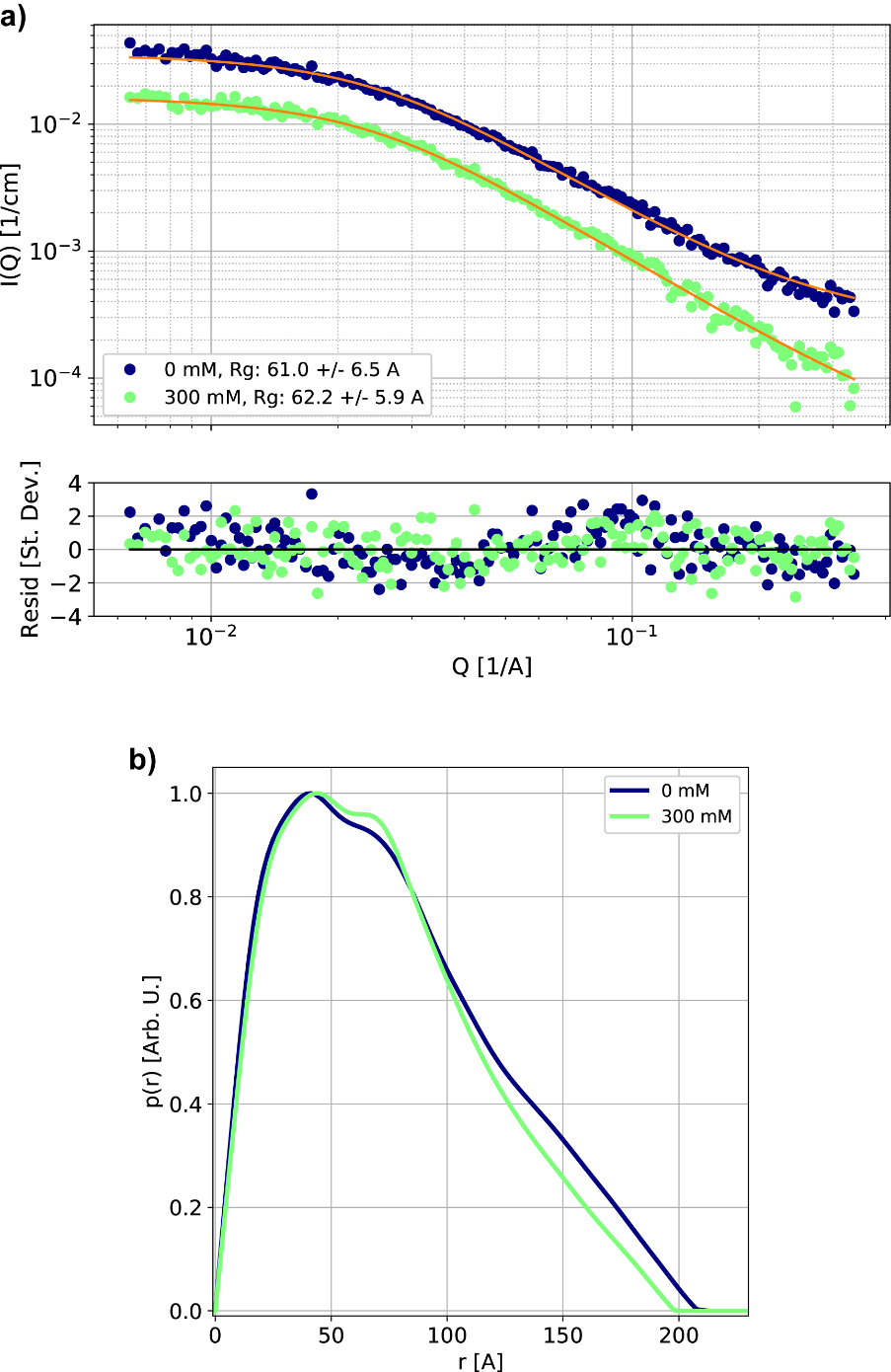
**

**Fig. S6: Small-angle X-ray diffraction analyses of GHR-LF-ICD**. a) Experimental SAXS data (spheres) and Gaussian random coil model fit (lines) for GHR-LF-ICD with different concentrations of NaCl, with the residuals from the fit shown below in units of standard deviations. b) Pair distance distribution functions for the GHR-LF-ICD SAXS data. The color coding is the same as in a).

**Supplemental Tables**

**Table S1. The probability of proline *cis* conformation in PRLR-ICD isoforms**

The probabilities were predicted from the amino acid sequence and chemical shifts using Promega [2]. The residue numbers of the prolines are indicated in bold, neighboring residues by small capital letters (numbers include the signal peptide).

|  | **_F_P269_P_** | **_P_P270_V_** | **_V_P272_G_** | **_G_P274_K_** |
| --- | --- | --- | --- | --- |
| LF | 0.08 | 0.02 | 0.01 | 0.02 |
| SF1b | 0.10 | 0.02 | 0.01 | 0.02 |

**Table S2. Overview of SLiMs in C1CRs isoforms**. Gain and loss of SLiMs by unique sequences predicted using the Eukaryotic Linear Motif (ELM) resource for Functional Sites in Proteins

|  |  | STAT3/5 | 14-3-3 | PDZ-1 | PDZ-2 | PDZ-3 | TRAF2/6 |
| --- | --- | --- | --- | --- | --- | --- | --- |
| PRLR | 1,2 | +YLDP/YLEV/YFHA/YVEI/YLDP | +RSSYNHITD* | - | - | - | +SREE |
|  | 4 | +YLEV | +RKISSQGRL | - | - | - | - |
|  | 5 | +YLEV | - | - | - | - | - |
|  | 6,8 | - | - | - | - | - | - |
|  | 9 | - | +RCCVSTGLT | - | - | - | - |
| TPOR | 1 | +YWQQ/YLRD/YLPD | - | - | - | - | +TCEE |
|  | 2 | - | +RWSRTCKQA  +RLLTL* | - | - | - | +PRQRGD** |
| GCSFR | 1 | +YLVQ/YLRC | +RAVSTQP | - | - | - | +PSQE |
|  | 3 | +YLVQ/YLRC | +RAVSTQP | - | - | - | +PQSE  +PQSEDD** |
|  | 4 | +YLVQ/YFKD | +RAVSTQP | +ITSVL | - | - | - |
| IL-31Rα | 1,2,6,12 | +YLKN/YVTC/YLKN | - | - | - | - | +TGQE/SFEE |
| (GMLR) | 3,5 | - | +RILSSCPTS | +CPTSI | - | - | +TGQE |
|  | 9 | - | - | - | - | - | - |
|  | 10 | - | - | - | +ARYQA | - | - |
|  | 11 | - | - | - | - | +LDCAF | - |
| IL-12Rβ | 1,3 | +YFQQ/YFDL | - | - | +DSLML | - | +SLEE |
|  | 2 | +YFQQ | +RRHSCPWTG | - | - | - |  |
| IL-23R | 1 | +YKPQ/YKPQ/YVDO/YIPD | +RISLL | - | - | - | +SVEE |
|  | 2,5,6,7 | +YFPQ | +RISLL | - | - | - | - |
| LEPR | B | +YMPQ/YLGV | +RVSCP | - | +CDLTV | - | +TGEE |
| (OBR) | A | - | - | - | - | +RTDIL | - |
|  | C | +YLLT | - | - | - | - | - |
|  | D | - | - | - | - | - | - |
| IL-7Rα | 1 | +YVTM/YQNQ | - | - | - | - | +SNQE |
|  | 3 | - | +RKVSVF | - | +SVFGA | - |  |
| IL-9Rα | 1,2 | +YLPQ/YCAL | +RPWKSVA  +RVQTLA  +RSWTF* | - | - | - | +ALEE |
|  | 3 | - | - | - | +GPVCC | - | - |
| IL-5Rα | 1,5 | +YIEK | - | +EDSVF | - | - | - |
|  | 4 | - | - | +KSSVI | - | - | - |
| G-MCSFRα | 1,7,8 | - | - | - | - | - | +TPEE |
|  | 2 | - | - | +SESSL | - | - | - |

*Experimentally determined, +KCSTWPLPQis non-canonical positioned 6 residues N-terminal to the predicted site and likely part of binding site [3].

**Minor TRAF sites

**Supplemental data**

**Interpretation of the diagram of states and conformational properties**

IDPs have been classified into diagram of states with five compositional groups based on their fraction of positively charged residues (f^+^) and fraction of negatively charged residues (f^-^) [4–6]. These two global parameters are combined into two measures underlying a diagram of states: the fraction of charged residues (FCR = f^+^ + f^-^) and the net charge per residue (NCPR = f^+^ - f^-^). If both FCR and |NCPR| are below 0.25, the IDR fall into region 1 (R1) of globules and tadpoles. The charge deficiency of R1 results in a preference for intra-chain interactions and hence, compact, but still dynamic, heterogeneous conformational ensembles. However, it should be noted that sequences belonging to R1 have been shown to both take on extended and compact ensembles [5]. If the FCR is between 0.25 and 0.35 and the |NCPR| below 0.35, the IDR falls into the region 2 (R2) of boundary IDRs. These IDRs have an intermediate fraction of charged residues roughly equally distributed between f- and f+, and hence, the conformational ensemble is neither dominated by charged nor uncharged residues. For this reason, the conformational properties of these IDRs cannot be predicted from global composition alone. When the FCR is above 0.35 and the |NCPR| is below 0.35, the IDR fall into region 3 (R3) of strong polyampholytes, which are IDRs that are both highly positively- and negatively charged. These IDRs may take on expanded or hairpin-like structures, dependent on the patterning or mixing of their oppositely charged residues. When both the FCR and the |NCPR| is above 0.35, the sequence of the IDR is strongly dominated by either positively or negatively charged residues, and it falls into region 4 (R4) (highly negative) or region 5 (R5) (highly positive) of strong polyelectrolytes. These IDRs typically adopt highly expanded conformations due to the repulsive forces between the like charges of their chains. For sequences in R3, R4 and R5, the charge fraction and patterning are likely to have a major impact on the conformation, while for sequences of R1 and R2, other residues may be more influential [5].

**Supplemental references**

1. Cheng Y, LeGall T, Oldfield CJ, Dunker AK, Uversky VN. Abundance of intrinsic disorder in protein associated with cardiovascular disease. Biochemistry. American Chemical Society; 2006;45:10448–60.

2. Shen Y, Bax A. Prediction of Xaa-Pro peptide bond conformation from sequence and chemical shifts. J Biomol NMR. 2010;46:199–204.

3. Olayioye MA, Guthridge MA, Stomski FC, Lopez AF, Visvader JE, Lindeman GJ. Threonine 391 phosphorylation of the human prolactin receptor mediates a novel interaction with 14-3-3 proteins. J Biol Chem. American Society for Biochemistry and Molecular Biology; 2003;278:32929–35.

4. Ruff KM. Predicting Conformational Properties of Intrinsically Disordered Proteins from Sequence. Kragelund BB, Skriver K, editors. Intrinsically Disord Proteins Methods Protoc Methods Mol Biol. Springer Science+Business Media, LLC, part of Springer Nature; 2020;2141.

5. Ginell GM, Holehouse AS. Analyzing the Sequences of Intrinsically Disordered Regions with CIDER and localCIDER. In: Kragelund BB, Skriver K, editors. Intrinsically Disord Proteins Methods Protoc Methods Mol Biol vol 2141. Springer Science+Business Media, LLC, part of Springer Nature 2020; 2020.

6. Das RK, Pappu R V. Conformations of intrinsically disordered proteins are influenced by linear sequence distributions of oppositely charged residues. Proc Natl Acad Sci. 2013;110:13392–7.
